# CTCF regulates the local epigenetic state of ribosomal DNA repeats

**DOI:** 10.1186/1756-8935-3-19

**Published:** 2010-11-08

**Authors:** Suzanne van de Nobelen, Manuel Rosa-Garrido, Joerg Leers, Helen Heath, Widia Soochit, Linda Joosen, Iris Jonkers, Jeroen Demmers, Michael van der Reijden, Verónica Torrano, Frank Grosveld, M Dolores Delgado, Rainer Renkawitz, Niels Galjart, Frank Sleutels

**Affiliations:** 1 Department of Cell Biology and Genetics, Erasmus MC, The Netherlands; 2 Department of Molecular Biology, Instituto de Biomedicina y Biotecnología de Cantabria, IBBTEC, Universidad de Cantabria-CSIC-IDICAN, Santander, Spain; 3 Institute for Genetics, Justus-Liebig-Universitaet Giessen, Heinrich-Buff-Ring 58-62, D-35392 Giessen, Germany; 4 Department of Reproduction and Development, Erasmus MC, The Netherlands; 5 Proteomics Center, Erasmus MC, The Netherlands; 6 Department of Epigenetics, Max-Planck Institute of Immunobiology, Freiburg, Germany; 7 Institute of Cell and Molecular Science, Centre for Gastroenterology, London, UK

## Abstract

**Background:**

CCCTC binding factor (CTCF) is a highly conserved zinc finger protein, which is involved in chromatin organization, local histone modifications, and RNA polymerase II-mediated gene transcription. CTCF may act by binding tightly to DNA and recruiting other proteins to mediate its various functions in the nucleus. To further explore the role of this essential factor, we used a mass spectrometry-based approach to screen for novel CTCF-interacting partners.

**Results:**

Using biotinylated CTCF as bait, we identified upstream binding factor (UBF) and multiple other components of the RNA polymerase I complex as potential CTCF-interacting partners. Interestingly, CTCFL, the testis-specific paralog of CTCF, also binds UBF. The interaction between CTCF(L) and UBF is direct, and requires the zinc finger domain of CTCF(L) and the high mobility group (HMG)-box 1 and dimerization domain of UBF. Because UBF is involved in RNA polymerase I-mediated ribosomal (r)RNA transcription, we analyzed CTCF binding to the rDNA repeat. We found that CTCF bound to a site upstream of the rDNA spacer promoter and preferred non-methylated over methylated rDNA. DNA binding by CTCF in turn stimulated binding of UBF. Absence of CTCF in cultured cells resulted in decreased association of UBF with rDNA and in nucleolar fusion. Furthermore, lack of CTCF led to reduced binding of RNA polymerase I and variant histone H2A.Z near the rDNA spacer promoter, a loss of specific histone modifications, and diminished transcription of non-coding RNA from the spacer promoter.

**Conclusions:**

UBF is the first common interaction partner of CTCF and CTCFL, suggesting a role for these proteins in chromatin organization of the rDNA repeats. We propose that CTCF affects RNA polymerase I-mediated events globally by controlling nucleolar number, and locally by regulating chromatin at the rDNA spacer promoter, similar to RNA polymerase II promoters. CTCF may load UBF onto rDNA, thereby forming part of a network that maintains rDNA genes poised for transcription.

## Background

CTCF is a conserved and ubiquitously expressed protein, which binds DNA through an 11-zinc finger (ZF) domain and organizes chromatin into loops [1]. CTCF may act as an insulator, mainly by inhibiting inappropriate interactions between regulatory elements on adjacent or distal chromatin domains. In many instances, CTCF binds cognate sites in a methylation-sensitive manner, allowing the regulation of imprinted loci, such as the *H19/Igf2 *locus. A testis-specific paralog of CTCF has been characterized, called CTCFL or BORIS (brother of the regulator of imprinted sites), which has strong similarity to CTCF in the ZF domain and has overlapping DNA-binding specificity [2]. CTCF and CTCFL share little similarity outside their ZF region. To date, no common interaction partners of CTCF and CTCFL have been reported.

Genomewide studies have revealed a multitude of CTCF binding sites, whose distribution over chromosomes correlates with gene density [3]. The cohesin complex, which mediates sister chromatid cohesion in dividing cells, was shown to colocalize with CTCF on CTCF binding sites [4-6]. Recent data suggest that CTCF/cohesin are together involved in the organization of chromatin loops, with CTCF recruiting cohesin to specific sites, and cohesin in turn mediating chromosomal interactions [7]. CTCF may also colocalize with the variant histone H2A.Z [8]. When CTCF is bound near an RNA polymerase II-regulated transcription start site (TSS), it is mostly located upstream of a DNAse I hypersensitive site (HS) which in turn precedes the TSS [9]. These data suggest a global role played by CTCF as an organizer of RNA polymerase II-mediated transcription. By contrast, we have shown that loss of a CTCF-binding site affects chromatin looping and local histone modifications in the mouse β-globin locus, without significantly perturbing transcription [10]. Collectively, these data indicate that CTCF is able to regulate the balance between active and repressive chromatin modifications near its binding sites, with different outcomes in terms of transcription. CTCF may control epigenetic modifications by binding to the chromatin remodeling factor CHD8 [11].

The nucleolus is a nuclear subcompartment in which the 18S, 5.8S and 28S ribosomal (r)RNAs are synthesized by RNA polymerase I, processed and, together with 5S rRNA, assembled into ribosomes [12]. Ribosome biogenesis is tightly coordinated with cellular metabolism and cell proliferation. In all organisms, ribosomal genes are repeated many times, so that enough rRNA can be produced when demand for ribosomes is high. However, even in metabolically active cells, a significant number of repeats are not transcribed. In human and mouse, there are approximately 200 rDNA repeats per haploid genome (that is, ~400 per interphase nucleus). These are clustered in five nucleolar organizer regions (NORs), located on different chromosomes. Two promoters have been identified within the mouse rDNA repeat: the spacer promoter and the gene promoter. The spacer promoter is located upstream of the gene promoter within the intergenic spacer (IGS). Transcription from this promoter is thought to serve a regulatory function and gives rise to non-coding RNAs (ncRNAs or nc-rRNAs). Transcription from the gene promoter yields a ~13 kb (or 47S) ribosomal precursor RNA (pre-rRNA), which is processed in a complex manner into the mature 18S, 5.8S and 28S rRNAs.

Efficient transcription from the ribosomal gene promoter requires a multiprotein complex including selectivity factor (SL)1, RNA polymerase I, and upstream binding factor (UBF) [13]. UBF is an abundant nucleolar protein that contains several HMG domains involved in DNA binding [14]. UBF binds dynamically throughout the rDNA repeat [15], and not only plays a role as a transcriptional activator of RNA polymerase I, but also in transcription elongation [16] and in the maintenance of the specific chromatin structure of NORs [17]. More recent data suggest that UBF is involved in determining the number of active rDNA genes [18].

To better understand the function of CTCF, we performed a screen for CTCF-interacting proteins. We found that both CTCF and CTCFL interact directly with UBF. CTCF binds immediately upstream of the ribosomal spacer promoter in a methylation-sensitive manner, and activates spacer promoter transcription. CTCF binding controls the loading of UBF onto rDNA, and the binding of RNA polymerase I and H2A.Z near the spacer promoter. Our data show that CTCF regulates the local epigenetic state of the rDNA repeat. CTCF may organize RNA polymerase I and II promoters in a similar manner. We propose that CTCF binding maintains rDNA repeats in a state poised for activation.

## Results

### Characterization of biotinylated CTCF

**Figure 1:**
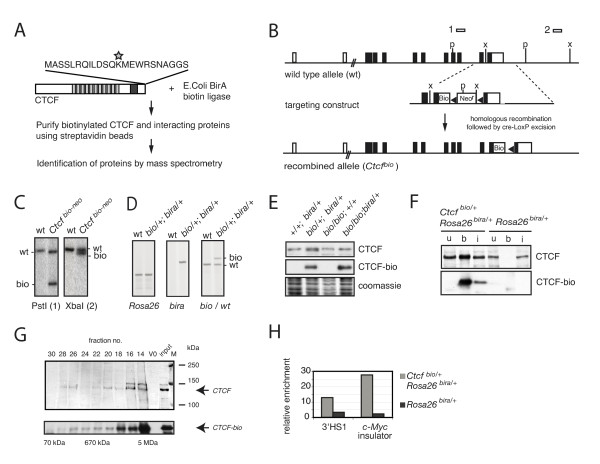
**Characterization of biotinylated CCCTC binding factor (CTCF)**. **(A) **Identification of CTCF-binding partners. CTCF (light grey boxes) was tagged with biotin at its C-terminus (biotinylated lysine indicated by a star) and pulled down, and interacting proteins were identified by mass spectrometry. Zinc fingers are indicated by dark grey box; proline-rich region with AT hook. **(B) **Targeting of mouse *Ctcf*. The *Ctcf *gene is shown on the top line. Exons (black boxes are coding, open boxes are non-coding) and probes (1, 2) are indicated. X = *Xba*I, P = *Pst*I. Targeting construct is shown in the middle. Bio = biotin-tag, black triangles = loxP sites, Neo^r ^= PMC1-neomycin cassette. After targeting, *Ctcf*^*bio-neo *^was obtained. Cre-mediated excision yielded the *Ctcf*^*bio *^knock-in allele (bottom line). **(C) **Southern blot analysis. Digested embryonic stem (ES) cell DNA was analyzed by Southern blotting using probes 1 or 2. Alleles are indicated. **(D) **PCR analysis of mouse tail DNA. **(E) **Expression of biotin tagged CTCF (CTCF-bio). Nuclear thymic extracts were analyzed by western blotting. Coomassie staining shows total protein. **(F) **CTCF-bio pull-down assay with streptavidin beads. Nuclear ES cell extracts were incubated with streptavidin-coupled magnetic beads. Input (i) = 5% of nuclear extract, (u) = 6% of unbound fraction, b = material bound to beads. **(G) **Size fractionation of CTCF and CTCF-bio. Size fractionated nuclear extracts were analyzed by western blotting. Molecular mass markers are indicated. V0 = void volume, input = nuclear extract (5%). **(H) **Chromatin immunoprecipitations. CTCF-bio precipitated with streptavidin-coupled magnetic beads from formaldehyde fixed nuclei bound known CTCF sites (β-globin 3" HS1 and c-Myc insulator).

To identify CTCF-binding partners, we used a biotinylation tagging and proteomics approach (Figure 1A) [19]. As CTCF levels are crucial for cell proliferation, we did not generate cell lines overexpressing biotinylated CTCF. Instead, we used homologous recombination in embryonic stem (ES) cells to generate a novel *Ctcf *knock-in allele. DNA encoding a small peptide tag of 23 amino acids was inserted in the last exon of the *Ctcf *gene, before the stop codon of CTCF (Figure 1B). This tag is biotinylated upon addition of the bacterial biotin ligase enzyme, BirA. Southern blot and PCR analysis identified homologous recombination events (Figure 1C). The resulting allele was termed *Ctcf*^*bio-neo*^, as it contains both the biotinylation sequence and the neomycin resistance gene.

*Ctcf*^*bio-neo/+ *^ES cells were transfected with a plasmid expressing Cre recombinase to remove the neomycin resistance gene and generate the *Ctcf*^*bio *^allele (Figure 1B). Then, using homologous recombination, the BirA biotin ligase was placed into the *Rosa26 *locus (data not shown). Genotyping and verification of these targeting events was performed by PCR (Figure 1D). This method yielded an ES cell line expressing normal CTCF (from the wild type allele) and biotinylated CTCF (from the *Ctcf*^*bio *^allele). The biotin tag is placed at the C-terminus of CTCF, hence the fusion protein was called CTCF-bio. *Ctcf*^*bio-neo *^ES cells were also injected into blastocysts to generate knock-in mice. These mice were subsequently crossed with a mouse line expressing BirA from the *Rosa26 *locus [20]. From these mice, CTCF-interacting proteins could be identified in a developmental and tissue specific manner.

CTCF-bio cannot be distinguished from untagged CTCF with anti-CTCF antibodies because the biotin tag does not cause a major difference in migration behavior in SDS-PAGE gels (Figure 1E, upper panel). However, CTCF-bio is detected using streptavidin-based methods (Figure 1E, middle panel). Our results indicate that CTCF-bio and CTCF are expressed at similar levels (Figure 1E). Pull-down assays using ES cell extracts with streptavidin-coupled magnetic beads results in efficient and specific binding of CTCF-bio to the beads (Figure 1F). Size fractionation experiments suggest that CTCF and CTCF-bio are present in high molecular weight complexes in ES cells (Figure 1G). Furthermore, CTCF-bio binds known CTCF target sites such as the *c-Myc *insulator and the 3" HS1 of *β-globin *(Figure 1H). Importantly, mice expressing CTCF-bio are viable and fertile (data not shown). Combined, these results indicate that CTCF-bio is a functional protein.

### CTCF and CTCFL interact with UBF

**Figure 2:**
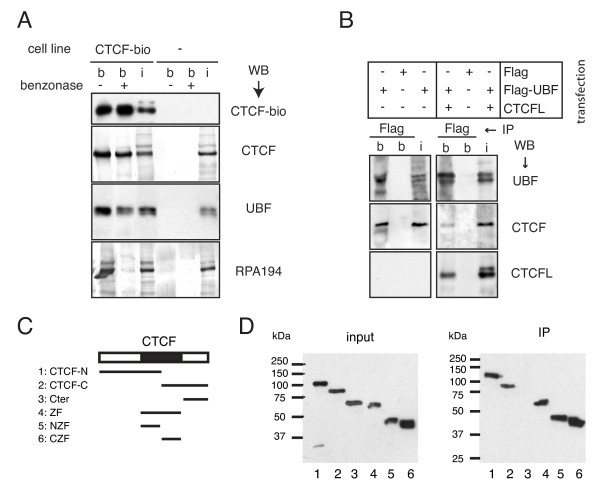
**Interaction of CCCTC binding factor (CTCF) and CTCFL with upstream binding factor (UBF)**. **(A) **Biotin tagged CTCF (CTCF-bio) interacted with UBF and RNA polymerase I in embryonic stem (ES) cells. Nuclear extracts from ES cells expressing CTCF-bio and control ES cells (-) were incubated with streptavidin beads, and CTCF-bio was purified with interacting proteins. Extracts were treated with (+) or without (-) benzonase for 2 hours at 4°C. Western blots were incubated with the indicated antibodies (CTCF-bio detected with streptavidin-coupled horseradish peroxidase). UBF was detected as a doublet consisting of UBF1 and UBF2; RPA194 = the large subunit of RNA polymerase I. B = bound fraction; i = input (5%). **(B) **Both CTCF and CTCFL interacted with UBF. Cells were transfected with different cDNAs (indicated below the lanes). CTCFL was not tagged. Immunoprecipitations (IPs) were carried out using anti-Flag antibodies. Western blots were incubated with antibodies against the indicated proteins. **(C) **Green fluorescent protein (GFP) tagged CTCF deletion mutants. The regions of CTCF used for making the different fusion proteins are indicated by lines. **(D) **UBF interacts with the zinc-finger domain of CTCF. GFP tagged CTCF deletion mutants (in (C)) were co-expressed with Flag tagged UBF in HEK293T cells. All fusion proteins are expressed at similar levels (input). After a Flag pull-down (IP), co-precipitating proteins were detected with an antibody against GFP. Lane numbers correspond to mutant numbers in (C).

CTCF-bio was purified from ES cell nuclear extracts under mild conditions using streptavidin-coupled magnetic beads (Figure 2A). Known CTCF-interacting partners, including Yin Yang (YY)-1, poly(ADP-ribose) polymerase (Parp)1 and nucleophosmin, co-precipitated with CTCF-bio (see Additional file 1, Figure S1A), further confirming that CTCF-bio is a functional fusion protein, and suggesting that the conditions used to isolate CTCF-bio were sufficiently mild to allow identification of novel interaction partners. Proteins co-purifying with CTCF-bio were detected by mass spectrometry and classified by BLAST searches; 58 of these co-purified specifically with CTCF in more than one pull-down experiment (data not shown). We noted that several CTCF-interacting proteins are involved in RNA polymerase I-mediated transcription (see Additional File 2 Table S1), including UBF and proteins that form a complex with UBF, such as the large subunit of RNA polymerase I (RPA194) and its associated factor PAF53 [21]. Moreover, the 40 kDa and 135 kDa subunits of RNA polymerase I (RPA40, RPA135) and polymerase associated factor (PAF)49 were pulled down by CTCF-bio (data not shown). These data suggest that CTCF interacts with essential components of the machinery that regulates the synthesis of rRNA. We therefore decided to further analyze the function of CTCF in rRNA transcription.

Streptavidin pull-down assays followed by western blot analysis confirmed the CTCF-bio interaction with UBF and the large subunit of RNA polymerase I (Figure 2A). We also detected interaction of CTCF-bio and UBF in lung (see Additional file 1, Figure S1B) and thymus (not shown), indicating that the association between these two proteins is not confined to ES cells. When ES cell nuclear extracts were treated with benzonase, the CTCF-bio interaction with UBF remained detectable, indicating that the interaction is not mediated by DNA (Figure 2A). Co-immunoprecipitation (co-IP) with anti-CTCF antiserum revealed an interaction between untagged CTCF and UBF (see Additional file 1, Figure S1C).

As CTCF and CTCFL are very similar in their ZF domains, we tested the possibility that CTCFL also interacts with UBF. We overexpressed a Flag-tagged form of UBF in 293T cells, either alone or with CTCFL, and performed a Flag co-IP on extracts from these cells. Flag-UBF brings down endogenous CTCF and overexpressed CTCFL (Figure 2B). Interestingly, diminished interaction between CTCF and UBF was detected in cells expressing CTCFL. These results identify UBF as the first common interaction partner of CTCF and CTCFL, and also indicate that CTCF and CTCFL compete for binding to UBF.

Experiments with bacterially purified proteins revealed a direct interaction between the CTCF and CTCFL ZF domains and the UBF dimerization domain plus HMG-box 1 (see Additional file 3, Figure S2). Using CTCF deletion mutants [22], we observed that both the N- and C-terminal ZFs of CTCF interacted with UBF (Figure 2C, D). Taken together, our data show that CTCF and CTCFL bind UBF directly via their ZF domains.

### Identification of a CTCF binding site upstream of the rDNA spacer promoter

**Figure 3:**
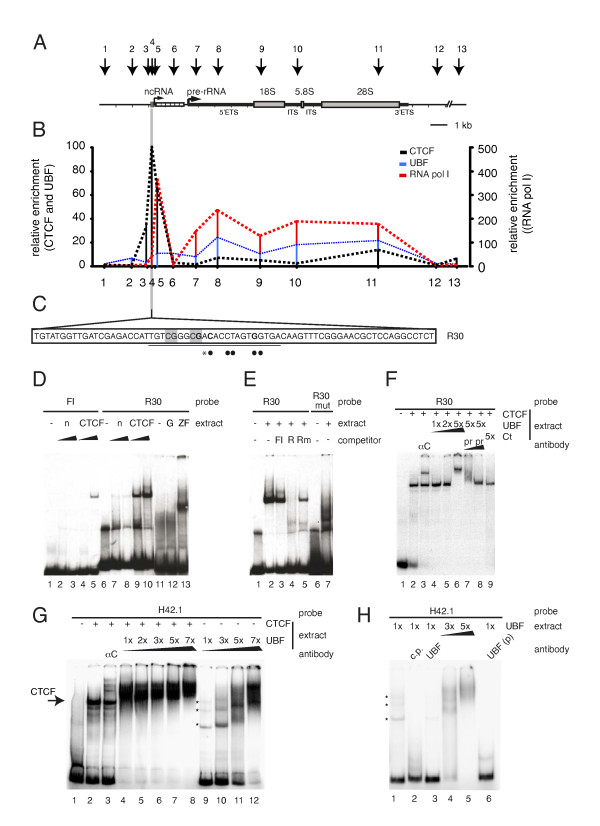
**Identification of a novel CCCTC binding factor (CTCF) binding site near the ribosomal (r)DNA spacer promoter**. **(A) **Outline of mouse rDNA repeat. Transcription (spacer promoter = ncRNA, gene promoter = pre-rRNA, right-pointing arrows), chromatin immunoprecipitation (ChIP) primers (downward-pointing arrows), organization of 47S pre-rRNA (external transcribed spacers (ETS), internal transcribed spacers (ITS), 18S, 5.8S and 28S rRNA, CTCF consensus site (grey box) and enhancer repeats (white boxes)), are indicated. **(B) **Binding of CTCF, upstream binding factor (UBF) and RNA polymerase I to mouse rDNA was analyzed by ChIP on formaldehyde-fixed mouse embryonic fibroblast (MEF) nuclei. **(C) **CTCF binding site in mouse rDNA. The R30 sequence is shown, with CTCF consensus site (underlined), highly conserved CTCF site residues (dots), deviation from consensus (asterisk) and CpG dinucleotides (gray). Nucleotides in bold were mutated (R30mut). **(D, E) **Band-shift analysis. Increasing amounts of HEK293T extracts (non-transfected (n) or transfected with CTCF), were incubated with the indicated probes (a known CTCF binding site (F1, chicken lysozyme, R30 and R30mut). Competitor was added in 300-fold excess. Band shifts were also performed with purified bacterial proteins (glutathione-S-transferase (GST) only (G) and zinc fingers (ZF) of CTCF tagged with GST. **(F) **CTCF and UBF bound R30 DNA together. HEK293T extracts (increasing amounts indicated, transfected with GFP-CTCF (+) Flag-UBF (1 to 5×) or the C-terminal domain of CTCF tagged with GFP (Ct, see Figure 2C) were incubated with R30. αC = addition of anti-CTCF; pr = addition of anti-UBF and anti-FLAG (preclear). **(G) **CTCF and UBF bound human rDNA together. HEK293T extracts (increasing amounts indicated, transfected with GFP-CTCF (+) or Flag-UBF (1 to 7×) were incubated with H42.1. Asterisks indicate UBF binding to H42.1. αC = addition of anti-CTCF. **(H) **UBF weakly bound human rDNA. HEK293T extracts (increasing amounts indicated), transfected with Flag-UBF (1 to 5×) were incubated with H42.1. Asterisks indicate UBF binding to H42.1 Cp = cold probe; UBF = addition of anti-UBF; UBF(p) = addition of anti-UBF and anti-FLAG before incubation with probe (preclear).

To provide a functional explanation for the CTCF-UBF interaction, we tested binding of both proteins using chromatin immunoprecipitation (ChIP) in mouse embryonic fibroblasts (MEFs). Consistent with published experiments [15,23], UBF bound throughout the enhancer/promoter regions and transcribed portion of the mouse rDNA repeat, with hardly any enrichment in the IGS (Figure 3B, blue line). By contrast, ChIP of CTCF revealed a highly specific accumulation immediately upstream of the rDNA spacer promoter (Figure 3B, black line). We also detected CTCF binding to the rDNA spacer promoter region in extracts of adult thymus from wild type and CTCF-bio-expressing mice (see Additional file 4, Figure S3). The CTCF binding coincided with (and was adjacent to) RNA polymerase I enrichment (Figure 3B, red line). Strong RNA polymerase I association to the spacer promoter relative to the gene promoter has also been shown by others [24-26].

The ChIP experiments suggest the presence of a CTCF binding site near the spacer promoter of the mouse rDNA locus. An algorithm was devised to search for potential binding sites within this locus. One site (R30), which conforms to the CTCF consensus sequence [3], is present in the spacer promoter area (Figure 3C). A probe (also called R30) was designed and tested in band-shift analysis, using nuclear extracts of non-transfected cells and of cells overexpressing CTCF. The known chicken lysozyme F1 site was used as control. We detected binding of endogenous CTCF and bacterially purified glutathione-S-transferase (GST)-CTCF-ZF to the R30 probe (Figure 3D, lanes 6 to 10 and 11 to 13, respectively). Competition experiments indicated that CTCF bound the FI probe less efficiently than it did R30, (Figure 3E, lanes 3 and 4). These data demonstrate that CTCF binds R30 through its ZF domain.

Previous studies have shown that mouse, rat and hamster rDNA repeats share significant sequence similarity in the spacer promoter region of the IGS [27]. Rat and hamster rDNA also contain the CTCF binding site (Figure S4, Additional file 5). Based on alignment information, we mutated three residues within R30, and performed band-shifts with normal and mutant R30 probes. As shown in Figure 3E, CTCF bound less efficiently to mutant R30 (lanes 5 and 7). Combined, these results identify a novel CTCF binding site in the mouse rDNA repeat that is conserved in rat and hamster.

The IGS of the human rDNA repeat is completely divergent in sequence from the mouse IGS (see Additional file 6, Figure S5A to C) and the presence of a spacer promoter has not been accurately described. Nevertheless, we identified two potential CTCF binding sites in the rDNA repeat, which were 0.9 kb and 5.1 kb upstream of the ribosomal gene promoter (called H42.1 and H37.9, respectively, for their respective positions) (see Additional file 6, Figure S5B). ChIP analysis revealed occupancy of CTCF at both sites, although binding was more prominent in the region near H42.1 than near H37.9 (see Additional file 7, Figure S6A). As K562 cells express both CTCF and CTCFL, we also tested whether CTCFL could bind the human rDNA repeat. Protein-DNA complexes were immunoprecipitated with two different CTCFL antibodies. CTCFL bound both H37.9 and H42.1, with a preference for site H42.1 (see Additional file 7, Figure S6A). We also detected binding of UBF to these rDNA regions (see Additional file 7, Figure S6A) and using sequential (ChIP-reChIP) analysis, found that CTCF and UBF were present on the same rDNA repeats (see Additional file 7, Figure S6B).

The CTCF ChIP results were confirmed *in vitro *by electrophoretic mobility shift assay (EMSA) analysis (see Additional file 7, Figure S6C). Nuclear extracts from cells transfected with CTCF showed stronger binding to H42.1 and H37.9 rDNA probes compared with extract from mock-transfected cells. The specificity of the binding was shown by competition with unlabeled probes and by supershift assays using anti-CTCF antibody. Incubation with an anti-actin antibody, used as a negative control, did not produce supershifts (data not shown). Together, these results demonstrate that CTCF associates upstream of the gene promoter in human rDNA and suggest that CTCF and UBF are bound together to the rDNA.

We next tested whether the *in vitro *binding of CTCF to DNA influences binding of UBF. Extracts of cells transfected with GFP-CTCF or Flag-UBF were incubated separately or together with the H42.1 probe, and binding of CTCF and UBF was examined by EMSA (Figure 3G, H). Binding of UBF alone to the H42.1 probe resulted in a relatively weak signal (Figure 3G, lane 9; Figure 3H, lane 1; asterisks), that was specific for UBF (Figure 3H, lanes 2, 3, 6). Increasing the amount of UBF in the reaction eventually led to enhanced and cooperative binding of UBF (Figure 3G, lanes 10 to 12; Figure 3H, lanes 4, 5). Interestingly, binding of CTCF to H42.1 resulted in enhanced binding of UBF at much lower levels of this protein (Figure 3G, compare lanes 4 to 8 with 9 to 12). These data suggest that CTCF helps to load UBF onto rDNA.

### CTCF binds rDNA in a methylation-sensitive manner

The CTCF R30 binding site in the mouse rDNA repeat includes two CpG residues (Figure 3C), which might be methylated *in vivo*. The CpG residues are conserved in rat and hamster (see Additional file 5, Figure S4). As CTCF often binds DNA in a methylation-sensitive manner, we tested whether the *in vitro *methylation of these two sites in R30 affected CTCF binding. We found that this was the case to some extent, as CTCF bound the non-methylated R30 probe slightly more efficiently, and this probe was a better competitor than methylated R30 (see Additional file 8, Figure S7A).

**Figure 4:**
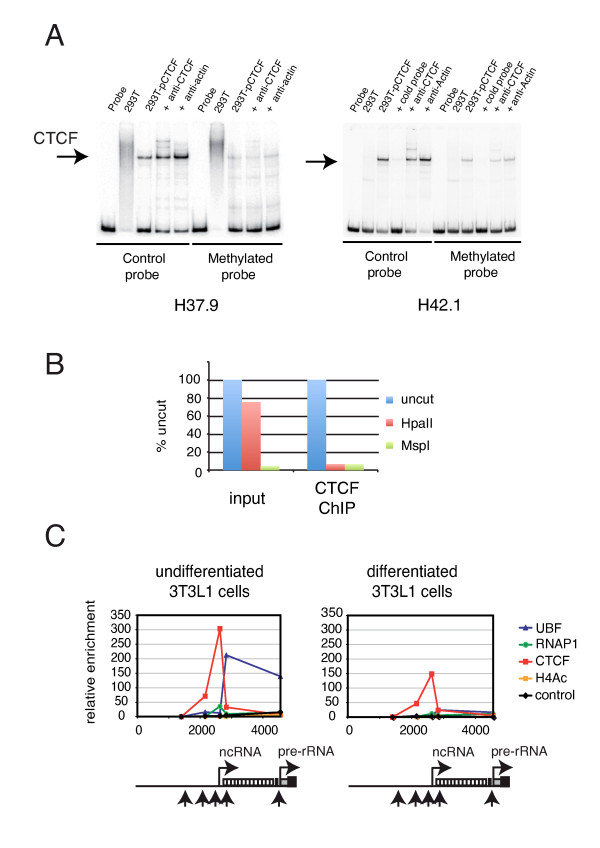
**CCCTC binding factor (CTCF) binding to ribosomal (r)DNA is methylation-sensitive**. **(A) **Influence of methylation on the binding of CTCF to rDNA. Band-shift assays using human H37.9 and H42.1 rDNA probes, either completely methylated with SssI methyltransferase (methylated probe) or non-methylated (control probe) on HEK293T extracts, transfected or not with CTCF. Competition was assessed by adding increasing amounts of non-labeled probe. In some cases, extracts were incubated with the indicated antibodies. **(B) **CTCF prefers non-methylated rDNA *in vivo*. Chromatin from K562 cells was immunoprecipitated with anti-CTCF (CTCF ChIP). Purified DNA was left uncut (mock digestion), or digested with *Hpa*II or *Msp*I. Quantitative PCR was then performed with H42.1 primers, both on non-precipitated K562 DNA (input) and on DNA enriched for CTCF binding sites (CTCF ChIP). Note the high content of *Hpa*II-resistant H42.1 rDNA in K562 cells (input), which represents methylated rDNA. In the CTCF-enriched sample, the *Hpa*II-resistant rDNA was not present, suggesting that CTCF does not bind well to methylated rDNA. **(C) **ChIP analysis on (left panel) undifferentiated and (right panel) differentiated 3T3L1 cells. Nuclei were fixed with 1% formaldehyde, and protein-DNA complexes were immunoprecipitated with antibodies against the indicated proteins (the large subunit of RNA polymerase I (RPA194), CTCF, upstream binding factor (UBF) and acetylated histone H4). The position of the primer sets (upward arrows, see also ChiP2 in Figure 6A), spacer promoter (right-pointing arrow with ncRNA (part A), enhancer repeats (white rectangles) and gene promoter (right-pointing arrow with pre-rRNA)) is indicated on the rDNA. The horizontal axis of the panels is co-aligned with the rDNA underneath and shows distance in base pairs.

Human 37.9 and 42.1 CTCF binding sites contain three CpG residues instead of two (not shown). One of these overlaps with the highly conserved GG dinucleotide that is part of the 'core' CTCF binding site (Figure 3C). The second CpG is conserved between human and mouse rDNA sites (it is the 5"end CpG in R30) (Figure 3C), whereas the third CpG in the human rDNA sites is not conserved between mouse and human, nor between 37.9 and 42.1 (not shown). We used SssI methyltransferase to completely methylate the human H37.9 and H42.1 probes (see Additional file 8, Figure S7B). Interestingly, CTCF binding to these fully methylated probes was severely reduced (Figure 4A). These data indicate that CTCF binds rDNA in a methylation-sensitive manner *in vitro*. Both the position and number of methylated CpG residues appear to influence CTCF binding to cognate sites.

To test whether DNA methylation might interfere with CTCF binding to the rDNA *in vivo*, we performed ChIP-chop experiments. Before quantitative PCR, the input and CTCF-enriched DNA samples were subjected to digestion with the methylation-sensitive enzyme *Hpa*II or the methylation-insensitive enzyme *Msp*I. CTCF did not bind *Hpa*II-resistant (that is, methylated) H42.1 rDNA (Figure 4B). Similar data were obtained for H37.9 rDNA (not shown). These results indicate that CTCF prefers non-methylated over methylated ribosomal DNA *in vivo*. A ChIP-chop assay performed on mouse ES cell DNA also showed CTCF binding to non-methylated rDNA (data not shown).

Fully methylated rDNA repeats are thought to be inactive [28]. To investigate an *in vivo *correlation between CTCF binding in the spacer promoter and methylation status of the rDNA repeats, we used 3T3L1 cells. These cells can be differentiated into adipocytes, which results in the repression of rRNA transcription by more than 50% [29]. Increased heterochromatin features at the rDNA promoter accompany this repression [29,30]. ChIP analysis revealed binding of CTCF, UBF and RNA polymerase I at the spacer promoter in undifferentiated 3T3L1 cells (Figure 4C, left panel). As reported previously [29], UBF and RNA polymerase I binding to the rDNA repeat was reduced in differentiated 3T3L1 cells (Figure 4C, right panel). CTCF binding was also significantly reduced (Figure 4C, right panel). These data suggest that increasing heterochromatinization *in vivo *significantly affects binding of CTCF, UBF and RNA polymerase I. We propose that *in vivo *CTCF binds rDNA repeats in a methylation-sensitive manner.

### CTCF regulates nucleolar number, and is required for UBF and RNA pol I binding near the spacer promoter

**Figure 5:**
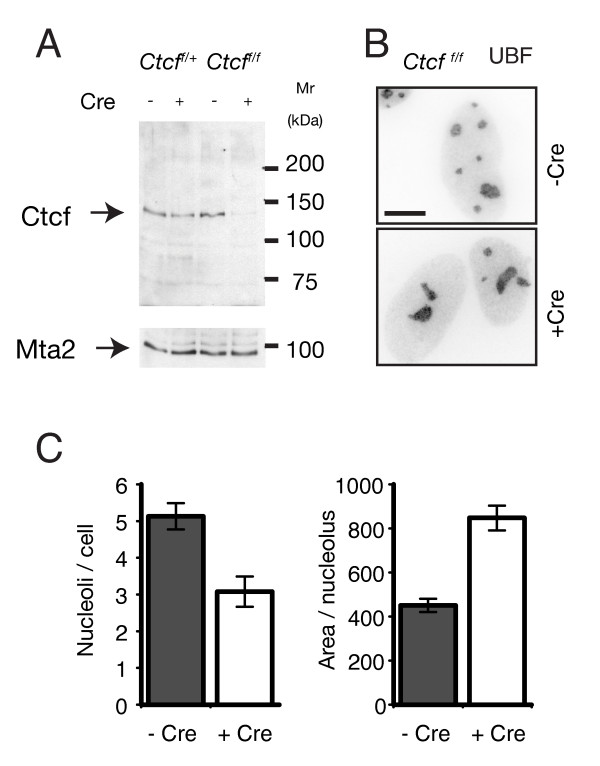
**CCCTC binding factor (CTCF) regulates nucleolar number**. **(A) **Efficient deletion of CTCF in mouse embryonic fibroblasts (MEFs). MEFs carrying the conditional *Ctcf*^*f *^knockout allele were infected (+) or not (-) with a lentivirus expressing Cre recombinase. After 4 days, cell extracts were analyzed for residual CTCF. Mta2 was used as loading control. In MEFs, *Ctcf *deletion with lentiviral Cre was very efficient, with > 90% of cells infected. **(B, C) **Distribution of upstream binding factor (UBF) in MEFs. Primary MEFs carrying the conditional *Ctcf*^*f *^knockout allele were either infected (+CrE) or not (-CrE) with a lentivirus expressing Cre recombinase. Cells were fixed, and incubated with antibodies against UBF (because both the CTCF and UBF antibodies used for immunofluorescence analysis were mouse monoclonals, we could not perform a combined CTCF/UBF stain. **(B) **Single image; **(C) **immunofluorescence results from several images were quantified.

To examine the physiological significance of a CTCF-UBF interaction and of CTCF binding to the rDNA spacer promoter, we generated a system to efficiently deplete CTCF *in vitro*. MEFs were isolated from mice homozygous for a *Ctcf *conditional knockout allele [31], and CTCF was deleted by infecting confluent MEFs with a replication-deficient lentivirus expressing Cre recombinase [10]. After 4 days of culture, only very low levels of CTCF protein were detected on western blot (Figure 5A). Immunofluorescence analysis revealed that a small proportion of MEFs still expressed CTCF (data not shown), suggesting that these were not infected by the virus. MEFs lacking CTCF could be maintained as a confluent layer for several days (data not shown), but they could not be passaged, because they are severely impaired in division. These results are in line with *in vivo *data showing that CTCF is essential for the proliferation and growth of β-selected T cells [31].

Next, we investigated the intracellular distribution of UBF in MEFs. Interestingly, deletion of CTCF reduced the number of UBF-positive spots, and thus the number of nucleoli, in MEFs (Figure 5B, C). However, the average size of a UBF-positive area, and thus that of a nucleolus, was larger in CTCF-deleted MEFs. As a result the total fluorescence intensity (and hence the level) of UBF was similar in CTCF-deleted and normal MEFs, a result supported by western blot analysis (not shown) and data in T cells [31]. We conclude that deletion of CTCF in MEFs results in fusion of nucleoli but does not affect UBF levels.

**Figure 6:**
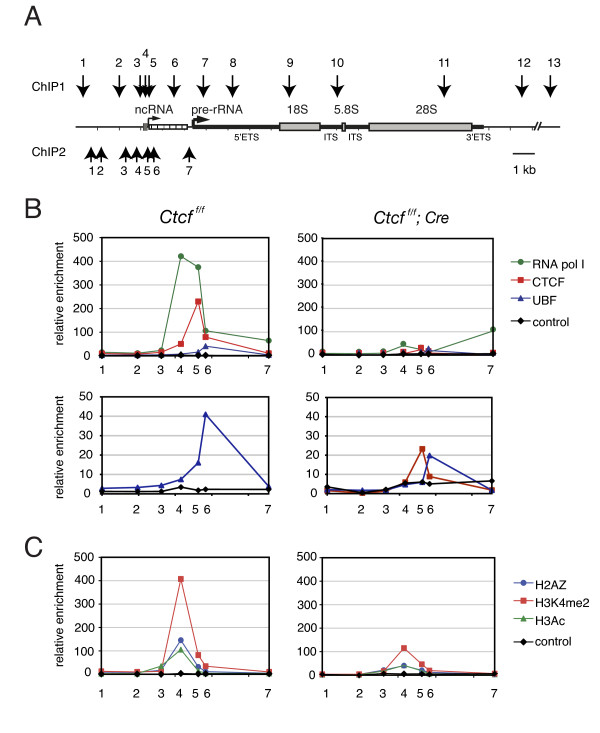
**CCCTC binding factor (CTCF) organizes chromatin on ribosomal (r)DNA**. **(A) **Outline of mouse rDNA repeat. The position of the primer pairs (1 to 7) used in the second chromatin immunoprecipitation (ChIP2; panel B) is indicated by upward-pointing arrows. For comparison, the primers used in the first ChIP (ChIP1; Figure 3) are also shown. For other explanations, see Figure 3A. **(B) **Binding of CTCF, upstream binding factor (UBF) and RNA polymerase I to the mouse spacer promoter. ChIP analysis was performed on mouse embryonic fibroblasts (MEFs) homozygous for the conditional *Ctcf *knockout allele (*Ctcf*^*f/*^^***(F)***^. MEFs were either (right panels) infected or not (left panels) with a lentivirus expressing Cre recombinase. Nuclei were fixed with 1% formaldehyde, and protein-DNA complexes were immunoprecipitated with antibodies against the indicated proteins (control = rabbit IgG). The upper and lower panels show the same results, but with a different vertical axis. Numbers on the horizontal axis refer to primer pairs. **(C) **Binding of modified and variant histones to the mouse spacer promoter. ChIP analysis was performed as described above. Protein-DNA complexes were immunoprecipitated with antibodies against the indicated proteins (control = rabbit IgG). Numbers on the horizontal axis refer to primer pairs.

CTCF binding to the rDNA spacer promoter was virtually undetectable in MEFs treated with Cre virus (Figure 6B, right-hand panels, red line) compared with non-treated MEFs (Figure 6B, left-hand panels, red line). In the absence of CTCF, binding of UBF and RNA polymerase I was severely reduced (Figure 6B, right hand panels, blue and green line, respectively). Remarkably, the absence of CTCF did not significantly perturb RNA polymerase I binding to the gene promoter. Thus, CTCF exerts a local influence.

In mouse ES cells, distribution of CTCF, UBF and RNA Pol I over the rDNA repeat, as analyzed by ChIP, was similar to that in MEFs (see Additional file 9, Figure S8). We used an RNA interference (RNAi)-based approach to knock down *Ctcf *mRNA in ES cells. Real time PCR and immunofluorescence analysis suggested knock down of CTCF of > 70% after 4 days of culture. Although the depletion of CTCF in ES cells was less effective than Cre-treatment of *Ctcf*^*f/f *^MEFs, this reduction did lead to a loss in UBF and RNA pol I binding (see Additional file 9, Figure S8B). These results confirm the role of CTCF in UBF and RNA polymerase I localization.

### CTCF maintains specific histone marks at the spacer promoter

Given the role of CTCF in epigenetic chromatin remodeling near its binding sites, we examined the distribution of specific histone marks across the rDNA regulatory region in the presence and absence of CTCF. ChIP analysis in normal MEFs revealed peaks of histone H3 acetylation, H3K4 dimethylation and H2A.Z just upstream of the CTCF binding site (Figure 6C, left panel). In the absence of CTCF H2A.Z, H3K4 dimethylation and H3 acetylation (that is, markers of 'active' chromatin and of insulator sites) were clearly downregulated (Figure 6C, right panel). A control ChIP experiment revealed similar amounts of histone H3 in the presence or absence of CTCF (see Figure S9A, Additional file 10), showing that the reduction in H2A.Z, H3K4me2 and H3ac levels is specific. Furthermore, ChIP analysis in the human rDNA repeat revealed specific accumulation of H2A.Z, H3K4me2 and H3ac at both CTCF binding sites in K562 cells (see Figure S9C, Additional file 10). Combined, our data suggest that CTCF is required for local histone modifications and the accumulation of a histone variant at the spacer promoter.

Because we found that CTCF is required for H2A.Z accumulation at the rDNA spacer promoter, we tested whether this also occurs with H2A.Z sites near RNA polymerase II-dependent genes. In the absence of CTCF, H2A.Z was indeed lost from the *c-Myc *promoter (see Additional file 10, Figure S9B), implying that CTCF can mediate the deposition of this histone variant close to RNA polymerase I and II promoters. The relatively constant levels of histone H3 in the rDNA locus in normal and Cre-treated MEFs (see Additional file 10, Figure S9A) indicate that the observed loss of histone modifications and H2A.Z are not caused by a reduction in nucleosomes in the absence of CTCF. Furthermore, the changes in DNA binding by specific proteins (, for example, UBF and RNA polymerase I) seen in the absence of CTCF, are not the result of changes in the total amount of these proteins (data not shown).

### CTCF activates transcription from the spacer promoter

**Figure 7:**
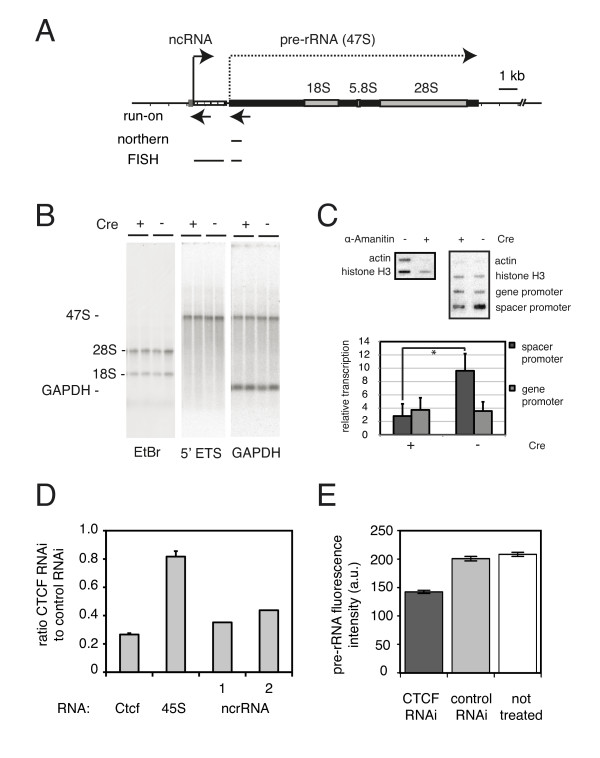
**CCCTC binding factor (CTCF) activates spacer promoter transcription**. **(A) **Transcribed portion of mouse ribosomal (r)DNA. Transcription initiation (spacer promoter = ncRNA, gene promoter = pre-rRNA, right-pointing arrows) and positions of run-on (left-pointing arrows), northern blot and fluorescent *in situ *hybridization (lines) probes are indicated. **(B) **Northern blot analysis. Total RNA from *Ctcf*^*f/f *^primary mouse embryonic fibroblasts (MEFs) (either treated (+) or not (-) with Cre) was analyzed sequentially with probes against the 47S pre-rRNA [32] and *Gapdh *(two samples per genotype shown, > 10 independent samples per genotype analyzed. **(C) **Spacer promoter transcription in the absence of CTCF. Nuclear run-on of spacer and gene promoter in *Ctcf*^*f/f *^MEFs (Cre-treated (+) or not (-)). The suppression effect of α-amanitin on RNA polymerase II-mediated transcription was measured. Left panel shows *Actin *and *Histone H3 *genes; a typical run-on is shown on the right. The graph shows the relative transcription activity of spacer and gene promoter (three independent experiments; *P = 0.005, Student t-test). **(D, E) **CTCF knockdown in embryonic stem (ES) cells. Cells were transfected with a pSUPER plasmid to knock down CTCF (controls were either no transfection, or transfection with a control vector). After 4 days, cells were harvested for **(D) **RNA analysis by PCR, or fixed and analyzed by fluorescent *in situ *hybridization **(E)**. ncRNA was determined twice with independent primers (1, 2), *Ctcf *and pre-rRNA were determined four times (SD indicated). Taking the average of the four ncRNA experiments showed a reduction of spacer transcript in CTCF-depleted cells to 39 ± 22% (mean ± SD). **(E) **Quantification was performed with > 300 cells per treatment (SEM indicated).

We next examined the effect of a CTCF deletion on steady state RNA levels using total RNA isolated from *Ctcf*^*f/f *^MEFs that were either treated or not treated with Cre virus. Using northern blot analysis, we found similar ratios of pre-rRNA (47S) to *Gapdh *mRNA in CTCF depleted MEFs (Figure 7B). Furthermore, the ratio of mature 18S rRNA to *Gapdh *mRNA was comparable in normal and CTCF-depleted cells. These results indicate that a deletion of CTCF does not affect steady state rRNA amounts in confluent non-dividing fibroblasts. Using nuclear run-on analysis, we investigated transcription from spacer and gene promoters in the presence and absence of CTCF. Deletion of CTCF significantly reduced transcription from the spacer promoter but did not affect transcription from the ribosomal gene promoter (Figure 7C). These results show that CTCF can activate transcription from the spacer promoter independently of the gene promoter.

We next examined the influence of CTCF on rRNA biogenesis in a cell type other than MEF. For these experiments, we used mouse ES cells, because RNAi-mediated knockdown of CTCF is effective in these cells. We investigated spacer and gene-promoter derived transcription in the presence or absence of CTCF using real-time PCR and fluorescent *in situ *hybridization (FISH). For the latter, we used probes against spacer promoter-derived transcripts ('ncRNA' probe), together with a previously described probe ('pre-rRNA' probe [32]) that covers the unstable 5" end of the external transcribed spacer (see Figure 7A for position of the probes on the rDNA). RNA FISH experiments showed that both the ncRNA probe (green) and the pre-rRNA probe (red) were located inside ES cell nucleoli (see Additional file 11, Figure S10C-E). Whereas the pre-rRNA signal was detected at a relatively constant level in each ES cell nucleolus, the ncRNA signal varied with respect to intensity and number of spots (on average ~4 per cell). These FISH experiments indicate that only a small subset of the rDNA repeats express ncRNA, consistent with recently published data [26]. Remarkably, ncRNA spots were often located at the periphery of the nucleolus, whereas pre-rRNA was detected throughout (see Figure S10C, D, Additional file 11). Our results suggest that ncRNA and pre-rRNA transcription can be independently regulated in space and time inside the nucleoli of ES cells.

Real-time PCR analysis suggests that pre-rRNA levels are reduced in ES cells lacking CTCF (Figure 7D). We also quantified pre-rRNA levels by measuring the fluorescence intensity in FISH experiments in non-treated, control RNA-treated, and CTCF RNAi-treated ES cells. Consistent with the PCR data, the pre-rRNA transcript was mildly affected in cells knocked down for CTCF (Figure 7E). Thus, in ES cells, lack of CTCF resulted in a very mild reduction of pre-rRNA levels, to ~80% of control. By contrast, both real-time PCR experiments using two different primers sets (Figure 7D) and RNA FISH data (see Additional file 11, Figure S10F) showed that ncRNA levels were significantly decreased in ES cells lacking CTCF. Combined with the run-on analysis in MEFs (Figure 7C) these results strongly suggest that CTCF activates transcription from the spacer promoter, an activity that is independent of cell type.

## Discussion

We have identified UBF as the first common interaction partner of CTCF and CTCFL, emphasizing a role for these proteins in the organization of rDNA chromatin. It will be interesting to determine how CTCFL influences rRNA transcription *in vivo*, as in normal tissues this protein is expressed in a very restricted manner [2], whereas its expression is upregulated in various types of cancers [33]. The ZF domain of CTCF and CTCFL mediates the interaction with UBF. In CTCF, this domain has also been shown to be responsible for interaction with other proteins, including CHD8 [11]. Interestingly, a ZF-dependent, pan-nucleolar localization of CTCF was described in K562 cells, which correlated with poly (ADP-ribosyl)ation and growth arrest of cells [34]. The pan-nucleolar distribution of CTCF indicates that the protein must be bound to rRNA and/or nucleolar proteins in addition to rDNA. It is therefore not surprising that CTCF function is different in K562 cells compared with MEFs or ES cells. Post-translational modifications may alter the function, localization and interactions of CTCF in a cell type-specific manner. We conclude that the ZF-domain of CTCF is a versatile nucleic acid and protein-protein interaction surface, explaining why it is so conserved.

Previously, the *Xenopus laevis *rDNA repeat was reported to contain multiple weak CTCF binding sites near its spacer promoter [35]. Although the physiological significance for rDNA transcription was not investigated in that study, the result is consistent with our data. The importance of CTCF binding near the spacer promoter is emphasized by the observation that the mouse binding site is conserved in rat and hamster. Furthermore, we identified two CTCF sites in the human rDNA (H37.9 and H42.1, respectively) upstream of the gene promoter. We found a specific accumulation of H2A.Z, H3K4me2 and H3ac at CTCF binding sites in the human and mouse rDNA repeats. Interestingly, enrichment of the acetylated histones H3 and H4 and of TATA binding protein (TBP) was observed 100 bp upstream of site H42.1, whereas UBF accumulates 3' to this site [36]. Thus, despite the fact that the IGS regions of mouse and human are not conserved (see Additional file 6, Figure S5A to C), critical factors and chromatin modifications are similarly organized around CTCF binding sites in rDNA (see Additional file 6, Figure S5D). In fact, our data suggest that the local organization of chromatin at CTCF sites near the RNA polymerase I-regulated spacer promoter and near RNA polymerase II promoters [9] is also similar. First, CTCF binds ~200 bp upstream of the TSS in both types of promoters, and an HS is present between the TSS and CTCF binding site. Furthermore, H2A.Z and H3K4me2 accumulate ~200 to 300 bp upstream of the CTCF binding site. Enrichment of H2A.Z at CTCF binding sites appears to be a general phenomenon [8,9,37]. In RNA polymerase II promoters, H2A.Z and H3K4me2 marks are associated with active or 'poised' promoters. We propose that binding of CTCF to the spacer promoter also maintains rDNA repeats 'poised' for transcription.

With one high affinity binding site per mouse rDNA, and with CTCF preferring non-methylated (and thus active) rDNA repeats, it is expected that only a small number of DNA-bound nucleolar CTCF molecules would be present. By contrast, UBF is abundantly present in the nucleolus, where it binds rDNA with low specificity [23] and is highly dynamic [15,38]. Thus, a UBF-CTCF interaction is expected to be transient. However, the interaction is crucial, as CTCF binding enhances UBF binding both *in vitro *and *in vivo*. Nucleolar UBF in turn ensures that rDNA repeats remain accessible to RNA polymerase I [18]. UBF, as part of the architectural HMG-box protein family, could change the topology of the rDNA, thereby facilitating binding of other factors [39], and allowing formation of small ~175 bp DNA loop structures called enhanceosomes [40]. In addition, CTCF and UBF may together recruit RNA polymerase I to the spacer promoter. Binding by CTCF to components of the RNA polymerase I complex would aid in this recruitment.

The biological function of the spacer promoter and the ncRNA transcript that is generated from it are still not completely understood. Early experiments suggested that the spacer promoter and the enhancer region act together to stimulate pre-rRNA transcription [27,41]. More recent data have shown that ncRNAs generated from the spacer promoter are unstable; transcripts are rapidly processed and degraded, and only the 3' end (~150 nucleotides) of the transcript, which matches the rDNA gene promoter, is bound to the nucleolar remodeling complex (NoRC) and is required for the establishment and maintenance of inactive rDNA repeats [24,30]. In this context, the spacer promoter transcript functions in rDNA silencing instead of activation.

Recent data implicate UBF in the balance between active and inactive rRNA genes, via a 'pseudosilencing' mechanism that is reversible and does not involve DNA methylation [18]. Thus, there appear to be two different epigenetic mechanisms that regulate the number of active rRNA genes. An attractive hypothesis is that CTCF, by binding to the spacer promoter of non-methylated (and thus active) rDNA repeats, and by interacting with UBF and 'loading' it onto these repeats, is involved in the 'pseudo-silencing' mechanism and maintains rDNA repeats 'poised' for transcription. At the same time, by generating spacer promoter transcripts, CTCF is 'feeding' NoRC with its 3' end degradation product, allowing this protein complex to function in a second epigenetic rRNA gene silencing mechanism. Consistent with this notion, ncRNA transcription appears to take place on a small subset of hypomethylated mouse rDNA repeats [26].

CTCF not only acts locally, but also regulates nucleolar number. Results in MEFs are consistent with data in T cells, where we found that the number of rDNA-positive signals decreases when CTCF is deleted *in vivo *[31]. Interestingly, B23 (or nucleophosmin) is a protein partner of CTCF, and B23-enriched insulator sequences are tethered to the nucleolar rim in a CTCF-dependent manner [42]. B23 is important for nucleolar structure [43]. Moreover, the borders of lamina-associated domains, detected by lamin B1, are demarcated by CTCF binding sites [44]. Lamin B1 interacts with B23, and is also involved in maintaining nucleolar structure [45]. We hypothesize that control of nucleolar number by CTCF is linked to its global function as an architectural factor, in association with proteins such as B23 and lamin B1.

Ribosome biogenesis controls cell growth and proliferation, as it determines the protein synthesis capacity of a cell. Recently, we showed that CTCF positively regulates cell growth in rapidly dividing thymocytes [31]. In the present study we detected multiple components of the RNA polymerase I complex in the mass spectrometry analysis of CTCF-bio-interacting partners. Knock-down of CTCF in ES cells resulted in slightly lowered levels of pre-rRNA. Conversely, under conditions of repressed pre-rRNA transcription, as in differentiated 3T3L1 cells [29], CTCF binding to the spacer promoter is reduced. Combined, these data suggest a link between CTCF, rRNA synthesis and cell growth control, whereby CTCF appears to act at a local and a global level.

## Conclusions

We show that CTCF and CTCFL bind UBF directly. CTCF organizes the local epigenetic state of rDNA repeats by regulating the binding UBF and RNA polymerase I and of other crucial components, and by altering chromatin modifications near its binding site. By tightly binding the rDNA spacer promoter, CTCF may enhance UBF binding and ensure that rDNA repeats are accessible to RNA polymerase I. CTCF binding at the spacer promoter stimulates transcription of non-coding RNA from the spacer promoter. The local organization of chromatin at CTCF sites near the RNA polymerase I-regulated spacer promoter and near RNA polymerase II promoters is remarkably similar. The CTCF-dependent enrichment of H2A.Z and H3K4me2 near the spacer promoter indicates that CTCF binding maintains rDNA repeats 'poised' for transcription.

## Methods

### Accession numbers and primers

We used mouse (accession number BK000964), human (U13369), rat (X04084) and hamster (DQ235090) rDNA sequences for alignments, to design primers for PCR and ChIP experiments and for probe generation. Primers used in all the different experiments are shown in Tables S2 tp S7 (Additional file 12, 13, 14, 15, 16, 17 respectively).

### Antibodies and cDNAs

CTCF mouse monoclonal antibodies were from BD Biosciences (Breda, NL), and CTCFL polyclonal rabbit antibodies (18337) were from Abcam. CTCFL (#4) polyclonal rabbit antibodies are described elsewhere (Sleutels *et al*, manuscript in preparation). The anti-CTCF (N3) and anti-RPA194 rabbit polyclonal antisera have been described previously [31,46]. Anti-histone H2A.Z (ab4174), anti-dimethyl-histone H3 (Lys4) (ab7766) and anti-histone H3 (ab1791) antibodies were from Abcam. Anti-acetyl histone H3 (06-599) and anti-acetyl Histone H4 (06-866) antibodies were from Upstate (Millipore, Amsterdam, NL). Anti-UBF (sc-13125) and anti-actin (sc-8432) antibodies were from Santa Cruz Biotechnologies (Santa Cruz, CA, USA). Streptavidin-HRP (RPN1231VS) and secondary HRP-labeled anti-mouse (NA931VS) and anti-rabbit antibodies (NA934V) were from Amersham (GE Healthcare, Uppsala, Sweden). Anti-His antibody was from Qiagen (Valencia, CA, USA), and anti-Flag M2 antibody was from Sigma Chemical Co. (St Louis, MO, USA).

His-tagged UBF fusion proteins were generated by PCR using mouse UBF cDNA from a Flag-tagged UBF construct as template (kind gift of Dr I. Grummt). Primers contained *Nhe*I and *Bam*HI sites for subcloning into the pET28a vector. GST-tagged fusions of mouse CTCF and CTCFL were amplified using mouse CTCF (IMAGE 6825952) and CTCFL (Sleutels *et al*, manuscript in preparation) cDNAs as templates. cDNAs were cloned into plasmid pGEX-3X and purified (glutathione-Sepharose 4B; Amersham Biosciences). GST-tagged fusion proteins derived from chicken CTCF have been described previously [47].

### Mass spectrometry

For mass spectrometry samples were treated and analyzed as described [48]. Data analysis and protein identification was performed as reported [49]. The Mascot search algorithm (version 2.0; MatrixScience) was used for searching against the NCBI database (taxonomy: *Mus musculus*). The Mascot score cut-off value for a positive protein hit was set to 60. Individual peptide tandem mass spectrometry (MS/MS) spectra with scores of < 40 were checked manually, and either interpreted as valid identifications or discarded. A number of CTCF-bio interacting proteins are listed in Table S1 (see Additional file 2). It should be noted that CTCF is difficult to purify under the mild conditions that are required to isolate associating proteins, although CTCF binds DNA tightly, the majority of its protein-protein interactions are of a transient nature.

### Affinity chromatography and size fractionation

Nuclear extracts were prepared as described previously [50]. Salt concentration in the extract was adjusted to 100 mmol/l NaCl. Unless stated differently, all IP and pull-down reactions were performed in IP buffer (100 mmol/l NaCl, 0.3% NP40, 20 mmol/l Hepes pH8, 0.2 mmol/l EDTA, 10 mmol/l MgCl_2_, with protease inhibitors) (Complete; Roche). Benzonase (Novagen) was added where indicated to remove DNA and RNA.

Streptavidin pull-down assays were performed as described previously [19], with the exception that the wash buffer and binding buffer were the same as the IP buffer described above. For IPs, nuclear extracts were pre-cleared at 4°C (Protein A sepharose beads, Sigma). Washes were performed at 4°C in wash buffer (100 mmol/l NaCl, 20 mmol/l Tris pH7.5, 0.3% NP40 and protease inhibitors). IPs were performed by adding antibodies to the samples and incubating for 1 hour at 4°C. Subsequently, protein-A sepharose beads were added, and incubation was continued for another hour at 4°C while rotating. Beads were washed six times with wash buffer.

Flag-IPs were performed using the same protocol as for IPs, except that anti-Flag M2 agarose (Sigma) incubation was performed for 3h at 4°C.

His-tagged proteins were bound to nickel-nitrilotriacetic (Ni-NTA) beads (Qiagen) in low salt buffer (20 mmol/l Hepes pH 7.5, 100mmol/l KCl, 10 mmol/l β-mercaptoethanol and 10% glycerol v/v). Proteins were purified by extensive washing of the beads, first in low-salt buffer, followed by washing in buffer with 1 mol/l KCl, and washing again in low-salt buffer. Proteins were eluted from the beads with 200 mmol/l imidazole in low-salt buffer, then the imidazole removed by dialysis. GST-tagged proteins were purified on glutathione-Sepharose 4B columns (Amersham Biosciences), using low and high salt buffers as above. To remove contaminating nucleic acids, benzonase was first added to bacterial extracts and again during washing of the (Ni-NTA) and glutathione beads. GST-based pull-downs were performed in binding buffer (20 mmol/l Tris-HCl pH 8, 100 mmol/l NaCl, 0.05% Triton X-100) containing benzonase, for 2 hours at 4°C. Washes were performed in binding buffer, followed by washes in high salt wash buffer (20 mmol/l Tris-HCl pH8, 400 mol/l NaCl, 0.05% Triton X-100). GST pull-downs on ES cell nuclear extracts were performed using the binding and washing conditions as described in the IP section.

Size fractionation of protein complexes was performed on a fast protein liquid chromatography apparatus (AKTA FPLC; Amersham Biosciences) with a Superose 6 10/30 column (Amersham Biosciences). Fractions were precipitated with 100% trichloroacetic acid and analyzed by western blotting as described previously [51]. Molecular size standards were thyroglobulin (670 kDa) and albumin (66 kDa) (Amersham Biosciences).

### SDS-PAGE, western blotting and EMSA

Bound proteins were eluted from beads by boiling in sample buffer (1 × Laemmli buffer). For western blot analysis, samples were separated by electrophoresis in SDS polyacrylamide gels and blotted onto poly(vinylidene fluoride membranes), (MilliPore) using a semi-dry blotting apparatus (BioRad). Signal detection was performed using enhanced chemiluminescence (Amersham).

For EMSA or band-shift analysis, protein extracts were preincubated with bandshift buffer (10% glycerol, 20 mmol/l Hepes pH7.4, 20 mmol/l KCl, 1 mmol/l MgCl_2_, 5 mmol/l dithiothreitol (DTT), 10 μmol/l ZnCl_2_, 100 μg/ml bovine serum albumin (BSA), 0.02% NP40) and 2 to 4 μg of salmon sperm DNA as a non-specific competitor. The reaction was incubated for 20 minutes at room temperature. Upon addition of probe the binding reaction was performed for another 20 minutes. Complexes were analyzed by electrophoresis through a 5% acrylamide (37,5:1) 0.5 × Tris/borate/EDTA non-denaturing gel at 8V/cm^2 ^at 4°C. Where specified, 300-fold excess of unlabeled probe or specific competitor was added at the same time as the probe.

Mouse probes for EMSA were end-labeled with ^32^P, whereas human probes (MYC-N, H42.1 rDNA and H37.9 rDNA) were ^32^P-labeled PCR fragments. For EMSA with *in vitro *methylated probes, purified H37.9 and H42.1 rDNA fragments (5 μl) were methylated *in vitro *using 12 U *Sss*I methyltransferase (New England Biolabs) and 1 μl S-adenosyl-L-methionine (32 mmol/l) in a final volume of 30 μl. Reactions were performed twice for 4 hours at 37°C, after which probes were purified. For supershift experiments, 1 μl of anti-CTCF mouse monoclonal or anti-actin (used as non-specific antibody) was added to the binding reaction before the radiolabeled probe.

### ChIP

Preparation of cross-linked chromatin (2 × 10^7 ^cells treated with 1% formaldehyde for 10 minutes at room temperature), sonication of chromatin to yield fragments of 300 to 800 bp, and immunoprecipitation were performed as described in the Upstate protocol http://www.upstate.com. At least two independent ChIPs were carried out per experiment. For streptavidin ChIPs, minor modifications were used: streptavidin beads were blocked for 1 hour at room temperature in 0.2 mg/ml sonicated salmon sperm DNA, elution was performed for 16 hours at 65°C in elution buffer (0.1% NaHCO_3_, 1% SDS, 0.2 mol/l NaCl). Quantitative real-time PCR (Opticon I, MJ Research and MyiQ, BioRad) was performed using SYBR Green (Sigma), Platinum *Taq *DNA polymerase (Invitrogen) and 100 ng of each primer under the following cycling conditions: 95°C for 3 minutes, followed by 40 cycles of 10 seconds at 95°C, 30 seconds at 60°C and 15 seconds at 72°C (during which measurements were taken). Values were normalized to input measurements, and enrichment was calculated relative to the *Amylase *gene using the comparative Ct method. PCR products were all < 150 bp.

For ChIP analysis with nuclei derived from human cell lines, 5 × 10^7 ^cells were fixed in 1% formaldehyde, lysed and sonicated. ChIP was performed using Dynabeads-protein G (Dynal Biotech) coupled to anti-CTCF, anti-CTCFL or anti-UBF antibodies. Dynabeads were incubated with lysates for 4 h at 4°C, and washed consecutively with commercial buffers (Low Salt, High Salt and LiCl Immune Complex Wash Buffers; Upstate). Chromatin was eluted with 200 μl of elution buffer (Upstate), de-crosslinked for 8 hours at 65°C, and purified (Qiaquick columns; Qiagen). Real-time PCR of immunoprecipitated DNA was performed with primers shown in Table S7 (see Additional file 17). The MYC-N and NY-ESO1 amplicons were used as positive controls for CTCF and CTCFL, respectively, and the MYC-H.1 amplicon as negative control. Enrichment for a specific DNA sequence was calculated as above.

### Methylation-sensitive ChIP assay (ChIP-chop)

To analyze the methylation density of rDNA precipitated with CTCF antibodies, post-ChIP hydrolysis ('chopping') of DNA was performed using the methylation sensitive enzyme *Hpa*II and its isoschizomer *Msp*I. Input sample (60 ng) and DNA from the CTCF ChIP reaction were divided into three equal aliquots, which were digested with *Hpa*II or *Msp*I, or left undigested (mock digested control). Digestions were carried out in a final volume of 20 μl for 3 hours at 37°C. Enzymes were inactivated for 30 minutes at 65°C. From each digestion, 10 μl was subjected to quantitative PCR with H42.1 rDNA primers, as described above. The uncut rDNA was set at 100%. The percentage of *Hpa*II and *Msp*I resistance was calculated as a percentage of mock digested DNA, by measuring the difference in Ct values in the qPCR (mock-*Msp*I or mock-*Hpa*II), taking the inverse of the fold difference in expression level, and multiplying this value by 100.

### Cell lines, transfections and lentiviral transduction

To generate the *Ctcf*^*bio-neo *^knock-in allele, a CTCF-TEV-bio in-frame fusion DNA was generated by PCR. In this construct, the biotinylation sequence [19] is preceded by a tobacco etch virus (TEV) protease cleavage site of seven amino acids. The neomycin-resistant LoxP-Neo-loxP vector and targeting procedures have been described previously [51]. IB10 129 ES cell DNA was analyzed by Southern blotting using radiolabeled probes outside of the region of homology (Figure 1A). For confirmation of homologous recombination, we used different 5' end and 3' end probes, and a PCR-based genotyping assay.

*Ctcf*^*bio-neo *^ES cells were transfected with CMV-Cre to remove the neomycin resistance cassette. A second round of homologous recombination was performed to target the Rosa26 locus with hemagglutinin (HA)-tagged BirA [20]. Verification of homologous recombined clones was performed by PCR. Control BirA-positive ES cell lines have been described previously [20].

3T3L1 cells (CL-173; ATCC) [29] and 293T cells [10] were cultured as described previously. The *Ctcf*^*f/f *^primary MEFs were isolated as described previously [51] at embryonic day 13.5 from embryos derived from conditional *Ctcf*^*f/f *^knockout mice [31].

Transient transfections in 293T cells with Flag-UBF and pcDNA3-CTCFL were performed using a transfection reagent (Lipofectamine™2000; Invitrogen) in reduced serum media (Optimem; GibcoBRL). Cells were analyzed 24 hours after transfection. Cre-lentivirus production and transduction of confluent primary MEFs was performed as described [10], with the exception that cells were split and diluted two-fold at 24 hours after transduction. Virus titers and Cre functionality were tested using serial dilutions. Recombination was tested after 4 days of infection by quantitative RT-PCR.

KCTCFD11 is a sub-line derived from K562 myeloid leukemia cells, which is stably transfected with a constitutive CTCF expression vector that moderately overexpresses CTCF (two to three-fold) compared with cells transfected with the empty vector (KpCDNA subline) [52]. For EMSA experiments, 293T cells or K562 cells were transfected with pcDNA3-CTCF expression vector (Lipofectamine™2000; Invitrogen).

### Northern blot analysis

Total RNA was isolated using an isolation solvent (RNA-Bee RNA Isolation Solvent; Tel-Test Inc.), size separated by gel electrophoresis (~6 μg per lane) and blotted onto membrane (Hybond N+; Amersham). Probes were radioactively labeled by PCR. Blots were exposed to screens (PhophorImager; Molecular Dynamics) to quantify results.

### Nuclear run-on

Cells were collected and washed twice with cold phosphate-buffered saline (PBS). The cells were lysed in nuclear isolation buffer (10 mmol/l Tris pH7.5; 10 mmol/l NaCl, 10 mmol/l MgCl_2_, 0.5% NP40). The nuclei were spun at 1000 g and resuspended in storage buffer (50 mmol/l Tris pH8.5, 0.1 mmol/l EDTA, 5 mmol/l MgCl_2_, 40% glycerol). Approximately 10^6 ^nuclei (50 μl) were pre-incubated for 20 minutes on ice with 100 μg/ml α-amanitin. Nuclei were then mixed with 50 μl 2 × reaction buffer (300 mmol/l KCl, 5 mmol/l MgCl_2_, 10 mmol/l Tris pH 7.5, 5 mmol/l DTT, 20 U RNA Guard, 0.5 mmol/l of each ATP, UTP and GTP, and 100 μCi of α^32^P CTP (800 Ci/mmol, 10 mCi/ml); Amersham). The labeling reaction was performed for 30 minutes at 30°C. The reaction was stopped on ice by adding 1 ml of isolation solvent (RNA-Bee) and total RNA was extracted as indicated above. Using a slot blot hybridization system with nylon membranes (Hybond-N+), 5 μg of DNA PCR fragments were hybridized with2×10^5 ^cpm of labeled RNA. Hybridization and detection was performed as described above. Incubation was performed in 2 ml of Church hybridization mix (0.5 mol/l Na_2_HPO_4 _pH 7.2, 7% SDS, 1 mmol/l EDTA) in a rotating hybridizer at 65°C for 24 h. Membranes were washed extensively at 65°C with Church wash buffer (40 mmol/l Na_2_HPO_4 _pH 7.2, 1% SDS). Hybridization signals were quantified with an imager (Phosphor Imager; Typhoon Amersham) using Imagequant software. The signal was corrected for the amount of CTG in the probe.

### Real-time PCR on ES cell RNA

Total ES cell RNA was isolated using Trizol (Invitrogen), treated with DNAseI, and converted into cDNA using random hexamers and reverse transcriptase (Superscript II; Invitrogen). Real-time PCR was performed using specific rRNA-covering primers and Sybr Green mix (Quantitect; Qiagen) on a performed on an automated PCR system (7500 Fast RT-PCR; Applied Biosystems). The negative control was as above with omission of the reverse transcriptase. The obtained Ct values were normalized to the Ct value of *Hprt*.

### FISH and immunofluorescence analysis

For FISH in ES cells, the cells were grown on coverslips. RNAi treatment of the cells was performed using a pSUPER vector-based system (CTCF RNAi sequence: 5"-GCAGAGAAAGTAGTTGGTAAT-3"). After transfection, cells were treated with puromycin to positively select for infected cells, thereby increasing the number of cells in which CTCF was knocked down. After 4 days of RNAi treatment, cells were fixed for 10 minutes with 4% paraformaldehyde (PFA) in PBS. Slides were stored in 70% ethanol until further use. For RNA FISH, cells were pretreated by two PBS washing steps, followed by a permeabilization step of 5 minutes in a solution of 25 μg/ml proteinase K in PBS. Slides were washed once in PBS, dehydrated and hybridized as described previously [53]. For DNA FISH, slides were pretreated by two PBS washing steps followed by a permeabilization step (4 minutes incubation in 0.1% pepsin in 0.01 mol/l HCl at 37°C). Slides were washed once in PBS on ice and fixed again for 5 minutes in 4% PFA in PBS. Slides were washed twice in PBS and dehydrated. Denaturation was performed for 2 minutes at 80°C in denaturing solution (70% formamide, 2 × saline sodium citrate, 10 mmol/l phosphate buffer pH 7), after which the slides were cooled in 70% ethanol, dehydrated, and hybridized as described previously [53].

The unstable 5" external transcribed spacers (ETS) probe has been described previously [32]. The enhancer probe used for DNA and RNA FISH (ncRNA; see Figure 7A for its position) was isolated as a 1.7 kb *Sal*I fragment from a cosmid covering a large part of the mouse rDNA repeat [32]. Probes were labeled by nick translation (Roche) using digoxygenin or biotin. Control DNA FISH experiments in ES cells showed that the ncRNA probe specifically localized to the nucleolus, as on pro-metaphase chromosomes the probe localized in discrete spots adjacent to centromeric DNA, indicative of NORs (see Additional file 11, Figure S10A), whereas in interphase cells the ncRNA probe localized within the nucleolus (see Additional file 11, Figure S10B). These data strongly suggest that the ncRNA probe specifically recognizes rDNA. When ES cells were treated with α-amanitin to inhibit RNA polymerase II transcription, both ncRNA and pre-rRNA signals remained visible (data not shown), confirming that RNA polymerase I is responsible for transcription of spacer and gene promoters.

For immunofluorescence staining, cells were fixed in 4% PFA in PBS for 15 minutes at room temperature, permeabilized in 0.15% Triton X-100 in PBS, blocked in 1% BSA in PBS and incubated with antibodies as described previously [32,51]. Images of cells were collected with a microscope (DMRBE; Leica) equipped with a camera (ORCA ER; Hamamatsu) or with a confocal lens (LSM510; Zeiss), as described previously [51].

For quantification of pre-rRNA signals, images of ES cells were collected with a microscope (DMRBE; Leica), using the same exposure time for all images. Five images each were collected of non-treated ES cells, control RNAi-treated ES cells and CTCF RNAi-treated ES cells. Collectively, more than 300 cells were present in the images, which were imported into Image J software (Rasband W.S., ImageJ, U. S. National Institutes of Health, Bethesda, Maryland, USA; 1997 to 2008; http://rsb.info.nih.gov/ij/,). Regions of interest (ROIs) were placed around individual pre-rRNA signals, using the freehand tool of Image J. ROIs were saved with the ROI manager. Both background fluorescence and mean fluorescent intensities of ROIs were calculated in each image. After deduction of the background fluorescence, mean fluorescence intensity data were collected into a spreadsheet (Excel; Microsoft), pooled and analyzed (Aabel software; GigaWiz). Quantification was performed in two independent experiments using different ES cell cultures, different probes and different RNAi treatments. Both experiments yielded similar results; that is, knock-down of CTCF leads to mildly reduced pre-rRNA levels.

## Competing interests

The authors declare that they have no competing interests.

## Authors' contributions

SvdN, MR-G, JL, FS and NG carried out specific experiments, participated in the design of the studies and helped to write the manuscript, HH, WS, IJ, JD, MvdR, VT and LJ carried out specific experiments, and participated in the design of the studies, FG, MDD and RR participated in the design of the studies, and helped to write the manuscript. All authors read and approved the final manuscript.

## Supplementary Material

Additional file 1 **Figure S1: Characterization of CCCTC binding factor (CTCF) and biotin tagged CTCF (CTCF-bio) interactions**. **(A) **CTCF-bio interacts with known CTCF binding partners. To identify CTCF-interacting proteins, CTCF-bio was purified from embryonic stem (ES) cell nuclear extracts under mild conditions. We validated our approach by showing that known interaction partners of CTCF, such as Yin Yang (YY)-1 [54], poly(ADP-ribose) polymerase (Parp)1 and nucleophosmin [42] co-precipitate with CTCF-bio. **(B) **CTCF-bio interacts with upstream binding factor (UBF) *in vivo*. Streptavidin pull-downs were performed using lung nuclear extracts isolated from mice expressing biotinylated (CTCF-bio) or normal (-) CTCF. Western blot analysis (b = bound fraction, i = input (5%)) revealed that CTCF-bio interacts with UBF. **(C) **Immunoprecipitation (IP) analysis of CTCF and UBF. IP was carried out on extracts of ES cells expressing both CTCF and CTCF-bio. We used specific antibodies against CTCF and UBF to precipitate endogenous proteins (IgG = control rabbit IgG). CTCF-bio was detected with horseradish peroxidase-coupled streptavidin. B = bound fraction, i = input (5%). Click here for file

Additional file 2 **Table S1: Mass spectrometry results for biotin tagged CTCF (CTCF-bio)**. Click here for file

Additional file 3 **Figure S2: Direct interaction of CCCTC binding factor (CTCF) and CTCFL with UBF**. **(A) **Schematic representation of the glutathione-S-transferase (GST)- and histidin (His)-tagged fusion proteins used. **(B) **Expression of GST- and His-tagged fusion proteins. Proteins were expressed in bacteria and affinity purified. Fusion proteins are indicated by asterisks. **(C) **Interaction between bacterially produced proteins. Purified GST- and His-tagged fusion proteins were incubated together, followed by GST pull-down. Western blots were incubated with an anti-His antibody. The experiments revealed a direct interaction between the CTCF and CTCFL zinc finger (ZF) domains and the upstream binding factor (UBF) dimerization domain plus high mobility group (HMG)-box 1. His-tagged proteins containing either the dimerization domain of UBF or HMG-box 1 only weakly bound CTCF and CTCFL, indicating that both regions are necessary for efficient interaction. **(D) **Bacterially produced CTCF and CTCFL interacted with UBF derived from embryonic stem (ES). GST pull-down assays of bacterially produced CTCF and CTCFL mutants were performed with nuclear protein extracts from ES cells. Equal amounts of GST fusion proteins were incubated with nuclear extracts from ES cells. Binding was performed under low-salt conditions, and washing was performed under more stringent conditions. Western blots were incubated with an antibody against UBF to detect ES cell-derived UBF. GST-tagged CTCF and CTCFL were both able to pull down specifically UBF. The ZF domains of CTCF (1) and CTCFL (5) displayed prominent interaction with ES cell-derived UBF. **(E) **Bacterially produced ZFP37 did not interact with histidine (His)-tagged UBF. The ZF domain of murine ZFP37 a protein that is enriched in the nucleolus [55] was tagged with GST. To provide further evidence for the specificity of the CTCF-UBF interaction, we examined whether this ZF domain interacts with UBF. Purified GST-tagged ZFP37 was incubated with His-tagged UBF (constructs 9, 10, 13). The interaction between CTCF (construct 1) and UBF was clear, but we could not detect any binding between UBF and ZFP37. Click here for file

Additional file 4 **Figure S3: Both CCCTC binding factor (CTCF) and biotin-tagged CTCF (CTCF-bio) bind the ribosomal (r)DNA spacer promoter *in vivo***. Extracts of adult thymus from wild type and Ctcf^bio/+^; Rosa26^bira/+ ^mice were analyzed for CTCF and CTCF-bio binding to the rDNA spacer promoter using anti-CTCF antibodies or a control serum (-). Click here for file

Additional file 5 **Figure S4: Comparison of mouse, rat and hamster ribosomal (r)DNA repeat regions**. Comparison of nucleotide sequences of the mouse, rat and hamster rDNA repeats [27]. Only the regions around the spacer promoter are indicated. Numbers to the left indicate distance (in base pairs) from the transcription start site of the gene promoter. The CCCTC binding factor (CTCF) consensus site [3] is underlined. Highly conserved CTCF consensus site residues are indicated by a dot (the asterisk indicates deviation between consensus site prediction and real residue). Conserved CpG dinucleotides are boxed. The transcription start site of the spacer promoter is indicated by a right-pointing arrow. Click here for file

Additional file 6 **Figure S5: Comparison of mouse and human rDNA repeat regions**. **(A-C) **Matrix plot comparisons of nucleotide sequences of **(A) **mouse versus mouse, (**(B) **human versus human and **(C) **human versus mouse rDNA repeats in the region upstream of the gene promoter. CCCTC binding factor (CTCF) binding sites are indicated (mCTCF BS for mouse, H37.9 and H42.1 for human). A highly repetitive Alu sequence is present ~2.5 kb upstream of the gene promoter of the human rDNA. Mouse rDNA does not have this repeat, but instead contains the well known 'enhancer repeat' region. Why CTCF binds twice in human and only once in mouse rDNA is unclear. One possibility is that CTCF has additional regulatory functions in the human rDNA repeat. For example, the H37.9 site is conserved in the rDNA of the great apes, as is the highly repetitive Alu repeat [56]. We speculate that H37.9 might be linked to the presence of this repetitive region in human and great ape rDNAs. **(D) **Similar chromatin organization of mouse and human rDNA repeat regions upstream of the gene promoter. The upper line represents the mouse rDNA (enhancer repeats are indicated by the open rectangles), and the lower line represents the human repeat. Only regions upstream of the gene promoter are shown. Right-pointing arrows indicate transcription from the gene promoter, giving rise to pre-rRNA. The spacer promoter has been clearly identified for the mouse but its location has not yet been mapped accurately for the human RNA. The chromatin organization surrounding the CTCF binding site (indicated by a lollipop) that is most proximal to the gene promoter, is strikingly similar in both mouse and human. In both organisms, the CTCF binding sites are embedded within a CpG island (as predicted with EMBOSS-CpG Plot [57]; the length of the CpG domains is indicated below the respective rDNAs). Immediately upstream of the CTCF binding site, mouse rDNA chromatin is enriched in 'active' histone modifications. A surprisingly similar result was previously obtained in the human locus (see Figure 5, site H42 in the paper by Grandori *et al. *[36]). Furthermore, TATA binding protein (TBP) has been shown to accumulate near the CTCF binding site, both in human [36] and mouse [25] rDNA repeats. We therefore propose that the spacer promoter in the human rDNA is located immediately downstream of the H42.1 CTCF binding site. Click here for file

Additional file 7 **Figure S6: CCCTC binding factor (CTCF) and CTCFL interact with human ribosomal (r)DNA *in vivo***. **(A) **Chromatin immunoprecipitation (ChIP) analysis on human rDNA. ChIP analysis with CTCF, CTCFL (two independent antibodies (Abs) were used) and UBF antisera, showing binding to the IGS of the rDNA repeat (sites H4, H37.9 and H42.1). Chromatin was prepared from non-transfected K562 cells or from cells stably transfected with CTCF (KCTCFD11) or the empty vector (KpCDNA). Relative enrichment was quantified by real-time PCR with the indicated primer sets. Known CTCF (MYC-N) and CTCFL (NY-ESO1) target sites were used as positive control for ChIP. Data were normalized against the enrichment for the negative control MYC-H.1. The value for the amount of PCR product present from the ChIP assay without antibody was set as 1 (white bars). Error bars represent the SEM of five to seven independent experiments for CTCF, eight to 10 for upstream binding factor (UBF), and four for CTCFL. **(B) **Sequential ChIP (ChIP-reChiP) analysis on human rDNA. Primary ChIP was performed as above, and CTCF or UBF ChIP products were subjected to a second immunoprecipitation (reChIP) with anti-UBF or anti-CTCF antisera, respectively. Relative enrichment was quantified by real-time PCR with primers for H37.9 or H42.1 rDNA, and data were normalized as in part (A). Error bars represent SEM of three independent experiments. Results show *in vivo *binding of CTCF and UBF simultaneously at rDNA sites. **(C) **CTCF interacts with human rDNA *in vitro*. Electrophoretic mobility shift assay (EMSA) analysis with nuclear extracts from 293T cells or K562 cells transfected with CTCF or mock transfected. ^32^P-labeled PCR fragments of MYC-N (positive control), H42.1 rDNA and H37.9 rDNA were used as probes. Unlabeled (cold) probes were used as competitors (Myc-N = 90% competition (compare lanes 1 and 3); H42.1 = 95% competition (compare lanes 5 and 7); H37.9 = 85% competition (compare lanes 11 and 13). Arrowheads indicate binding of CTCF; asterisks indicate supershift bands that appear after incubation with anti-CTCF antibody. Click here for file

Additional file 8 **Figure S7: CCCTC binding factor (CTCF) binds human ribosomal (r)DNA in a methylation-sensitive manner**. **(A) **Influence of methylation on the binding of CTCF to mouse rDNA. Band-shifts were performed using the mouse rDNA probe (R30) and the same probe methylated on two cytosine residues (R30-CmE). Competition was assessed by adding increasing amounts of unlabeled probe. **(B) **Control and SssI methyltransferase-treated H37.9 and H42.1 rDNA probes were digested with the methylation-sensitive enzyme *Hpa*II to assess the level of *in vitro *methylation. Fragments were separated in 8% polyacrylamide gels. Click here for file

Additional file 9 **Figure S8: Chromatin immunoprecipitation (ChIP) analysis in embryonic stem (ES) cells**. **(A) **Outline of mouse rDNA repeat. The position of the primer pairs used in the ChIP in panel B is indicated by downward-pointing arrows. Transcription initiation from the spacer promoter (yielding ncRNA) and the gene promoter (yielding pre-rRNA) is indicated by right-pointing arrows. The 47S pre-rRNA is divided into 5' and 3' external transcribed spacer (ETS), internal transcribed spacers (ITS), and 18S, 5.8S and 28S rRNA genes. The approximate positions of the CCCTC binding factor (CTCF) consensus site (gray box) and enhancer repeats (white boxes) are indicated. **(B) **ChIP assay on mouse rDNA. Binding of CTCF (black), upstream binding factor (UBF) (purple/blue) and RNA polymerase I (red) to mouse rDNA was analyzed using the primer pairs indicated in part (A). Embryonic stem (ES) cells were treated with control (straight lines) or *Ctcf *(stippled lines) RNAi constructs. ES cell nuclei were fixed with 1% formaldehyde, and protein-DNA complexes were immunoprecipitated with antibodies against the indicated proteins. Upon depletion of CTCF, binding of both RNA Pol I and UBF was diminished. Strikingly, for both proteins, loss in binding was greatest near the CTCF binding site, strongly suggesting an important role for CTCF in the binding of these proteins at or near the spacer promoter (RNA Pol I, UBF) and on the enhancer repeat (UBF). The fact that RNA Pol I binding was not affected at or downstream of the gene promoter is consistent with previous data. Click here for file

Additional file 10 **Figure S9: CCCTC binding factor (CTCF) regulates histone deposition**. **(A) **Binding of histone H3 to mouse ribosomal (r)DNA. Chromatin immunoprecipitation (ChIP) analysis is the same as shown in Figure 6, but a lower y-axis scale is used to demonstrate the histone H3 binding pattern. Enrichment was normalized to input and is shown relative to the *Amylase *gene (note that in this case the *Amylase *gene is not a negative control, because histone H3 will also bind this gene, hence the 'low' relative enrichment). Histone H3 was distributed in a similar manner in Cre-treated *Ctcf*^*f/f *^mouse embryonic fibroblasts (MEFs) compared with non-treated cells. Interestingly, binding appeared to diminish as the ribosomal gene promoter area is approached. This might be due to the fact that active ribosomal genes contain fewer nucleosomes [12]. **(B) **Binding of CTCF and H2A.Z to the *c-Myc *gene. ChIP analysis was performed as in Figure 6C, using the regulatory region upstream of the *c-Myc *transcriptional start site (SD of three independent experiments indicated). The position of the primer sets is indicated with arrows. **(C) **Binding of modified and variant histones to human rDNA. ChIP analysis was performed as in Figure S6A (see Additional file 7). Chromatin was prepared from K562 cells. Protein-DNA complexes were immunoprecipitated with antibodies against the indicated proteins. ChIP analysis showed specific binding of H2A.Z, H3ac and H3K4me2 to sites H37.9 and H42.1 of the rDNA. Click here for file

Additional file 11 **Figure S10: Spatial segregation of non-coding (nc)RNA and pre-rRNA transcription**. A, **(B) **DNA fluorescent *in situ *hybridization (FISH) analysis. The ncRNA probe (biotin-labeled, green) was hybridized to fixed and denatured ES cells. **(A) **Cell in prometaphase, with chromosomes condensed but not yet aligned. The ncRNA probe localized in distinct spots (arrows) adjacent to the strongly 4',6-diamidino-2-phenylindole (DAPI)-stained centromeric DNA. **(B) **An interphase cell, with the ncRNA probe localized to the nucleolus (visualized as weakly staining DAPI regions). Scale bars = (A) 2 μm, (B) 3 μm. **(C-F) **RNA FISH analysis. The ncRNA probe (biotin-labelled, green) and pre-rRNA probe (digoxygenin-labeled, red) were hybridized to fixed non-denatured ES cells. **(C-E) **Embryonic stem (ES) cells contain normal levels of CCCTC binding factor (CTCF), whereas **(F) **ES cells transfected with a pSUPER plasmid have CTCF knockdown. **(C) **Low resolution image of a small ES cell colony (cells in the middle are less well visualized because these cells grow in clumps). Multiple nuclei (one is outlined), particularly on the edge of the colony, had readily detectable ncRNA and pre-rRNA signals. Scale bar = 10 μm. **(D) **High resolution confocal image of a single DAPI-stained ES cell nucleus. Both ncRNA and pre-rRNA signals were localized exclusively to the three nucleoli present within this cell. Five ncRNA spots are visible (arrows), localized at the periphery of the nucleoli. Scale bar = 1 μm. **(E, F) **Confocal images taken with similar settings. **(E) **non-treated ES cells; **(F) **CTCF RNAi-treated ES cells. The ncRNA signal is indicated by arrows. Depletion of CTCF led to a reduction in ncRNA. ncRNA was lacking in many cells throughout a 3D confocal stack. In cells lacking ncRNA, pre-rRNA levels also seemed to be affected (see asterisks). Scale bars (E, F) = 8 μm. **(G) **Knock-down of CTCF in ES cells. ES cells were transfected with a pSUPER plasmid to knock down CTCF. After 4 days, < 50% of the cells expressed detectable levels of CTCF (red), as detected by immunofluorescent staining with anti-CTCF antibodies. Nuclei were stained with 4',6-diamidino-2-phenylindole (DAPI). By contrast, cells treated with a control RNAi vector all expressed CTCF (not shown). Scale bar = 8 μm. Click here for file

Additional file 12 **Table S2: Primers used for band-shift assays **[58]. Click here for file

Additional file 13 **Table S3: Primers used for genotyping**. Click here for file

Additional file 14 **Table S4: Primers used for mouse chromatin immunoprecipitation (ChIP)**. Click here for file

Additional file 15 **Table S5: Primers used for northern blotting and nuclear run-on assays**. Click here for file

Additional file 16 **Table S6: Primers used for real-time PCR on embryonic stem (ES) cell ribosomal (r)RNA**. Click here for file

Additional file 17 **Table S7: Primers used for human chromatin immunoprecipitation (ChIP) and band-shift assays **[59-61]. Click here for file
