# Supplementary figures and images for: CTCF regulates the local epigenetic state of ribosomal DNA repeats
Source: Epigenetics Chromatin. 2010 Nov 8;3:19. doi: 10.1186/1756-8935-3-19 (PMC2993708; doi:10.1186/1756-8935-3-19)

Figure S1- van de Nobelen et al

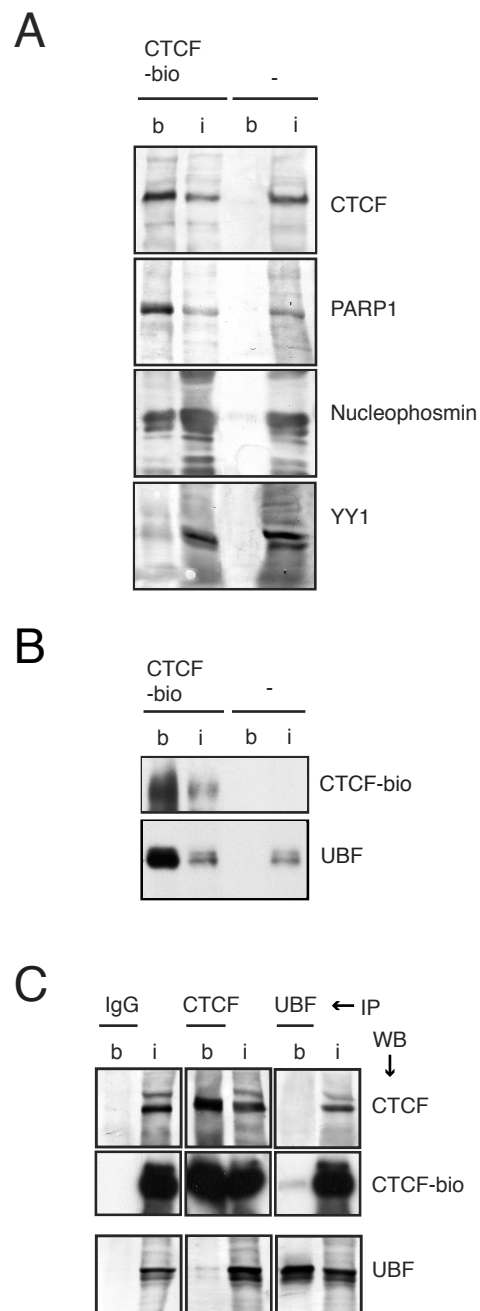

Supplement: Additional file 1 — Figure S1: Characterization of CCCTC binding factor (CTCF) and biotin tagged CTCF (CTCF-bio) interactions. (A) CTCF-bio interacts with known CTCF binding partners. To identify CTCF-interacting proteins, CTCF-bio was purified from embryonic stem (ES) cell nuclear extracts under mild conditions. We validated our approach by showing that known interaction partners of CTCF, such as Yin Yang (YY)-1 [54], poly(ADP-ribose) polymerase (Parp)1 and nucleophosmin [42] co-precipitate with CTCF-bio. (B) CTCF-bio interacts with upstream binding factor (UBF) in vivo. Streptavidin pull-downs were performed using lung nuclear extracts isolated from mice expressing biotinylated (CTCF-bio) or normal (-) CTCF. Western blot analysis (b = bound fraction, i = input (5%)) revealed that CTCF-bio interacts with UBF. (C) Immunoprecipitation (IP) analysis of CTCF and UBF. IP was carried out on extracts of ES cells expressing both CTCF and CTCF-bio. We used specific antibodies against CTCF and UBF to precipitate endogenous proteins (IgG = control rabbit IgG). CTCF-bio was detected with horseradish peroxidase-coupled streptavidin. B = bound fraction, i = input (5%). [file 1756-8935-3-19-S1.PDF]

Figure S3 - van de Nobelen et al

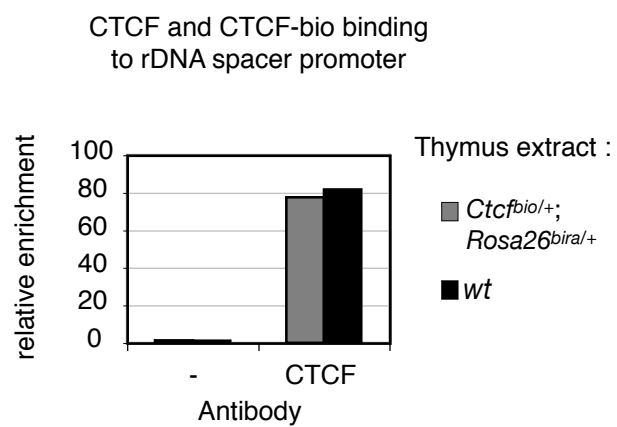

Supplement: Additional file 4 — Figure S3: Both CCCTC binding factor (CTCF) and biotin-tagged CTCF (CTCF-bio) bind the ribosomal (r)DNA spacer promoter in vivo. Extracts of adult thymus from wild type and Ctcfbio/+; Rosa26bira/+ mice were analyzed for CTCF and CTCF-bio binding to the rDNA spacer promoter using anti-CTCF antibodies or a control serum (-). [file 1756-8935-3-19-S4.PDF]

Figure S4 - van de Nobelen et al

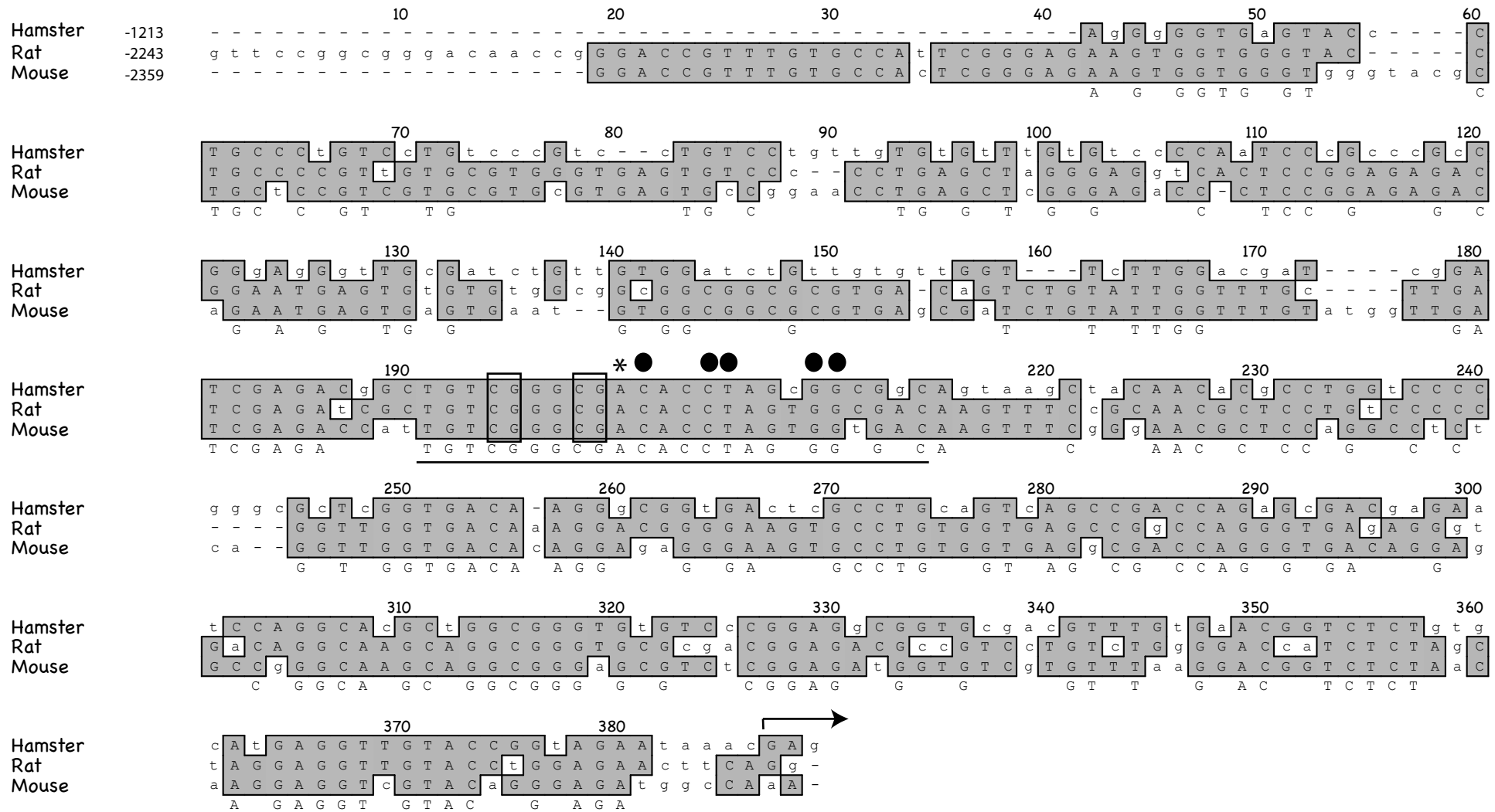

Supplement: Additional file 5 — Figure S4: Comparison of mouse, rat and hamster ribosomal (r)DNA repeat regions. Comparison of nucleotide sequences of the mouse, rat and hamster rDNA repeats [27]. Only the regions around the spacer promoter are indicated. Numbers to the left indicate distance (in base pairs) from the transcription start site of the gene promoter. The CCCTC binding factor (CTCF) consensus site [3] is underlined. Highly conserved CTCF consensus site residues are indicated by a dot (the asterisk indicates deviation between consensus site prediction and real residue). Conserved CpG dinucleotides are boxed. The transcription start site of the spacer promoter is indicated by a right-pointing arrow. [file 1756-8935-3-19-S5.PDF]

Figure S5 - van de Nobelen et al

A

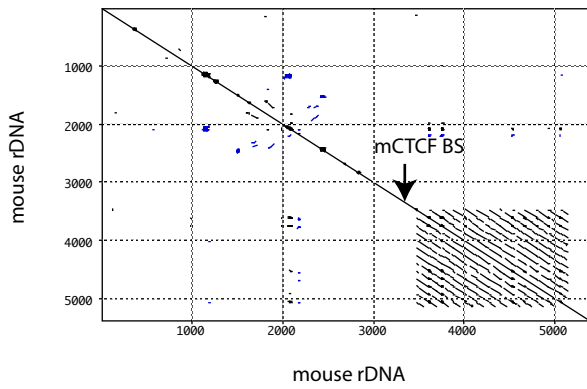

B

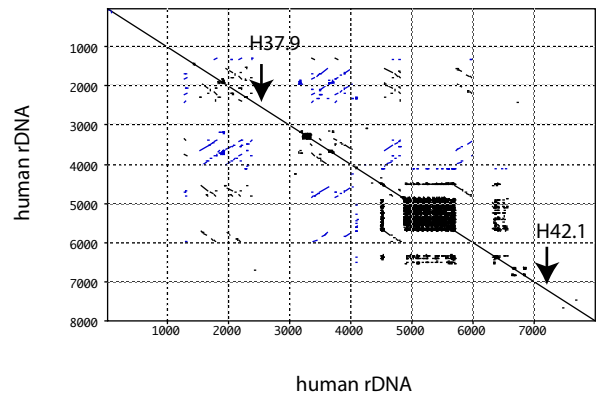

C

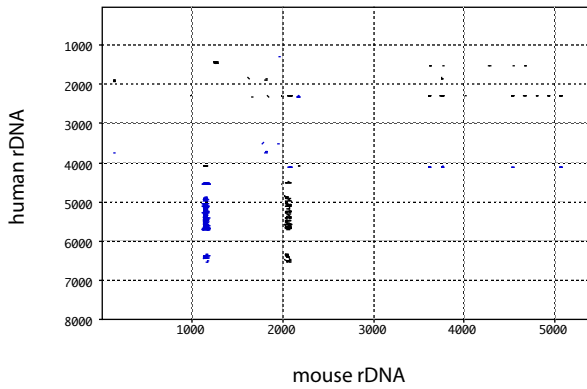

D

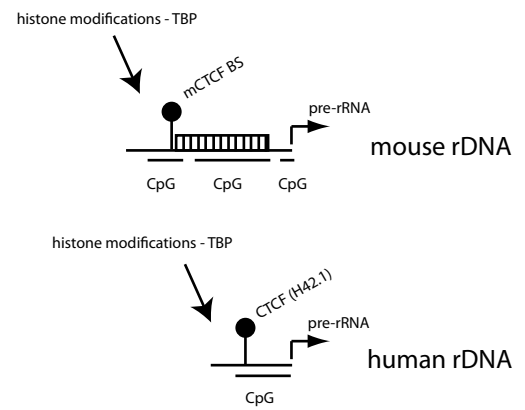

Supplement: Additional file 6 — Figure S5: Comparison of mouse and human rDNA repeat regions. (A-C) Matrix plot comparisons of nucleotide sequences of (A) mouse versus mouse, ((B) human versus human and (C) human versus mouse rDNA repeats in the region upstream of the gene promoter. CCCTC binding factor (CTCF) binding sites are indicated (mCTCF BS for mouse, H37.9 and H42.1 for human). A highly repetitive Alu sequence is present ~2.5 kb upstream of the gene promoter of the human rDNA. Mouse rDNA does not have this repeat, but instead contains the well known 'enhancer repeat' region. Why CTCF binds twice in human and only once in mouse rDNA is unclear. One possibility is that CTCF has additional regulatory functions in the human rDNA repeat. For example, the H37.9 site is conserved in the rDNA of the great apes, as is the highly repetitive Alu repeat [56]. We speculate that H37.9 might be linked to the presence of this repetitive region in human and great ape rDNAs. (D) Similar chromatin organization of mouse and human rDNA repeat regions upstream of the gene promoter. The upper line represents the mouse rDNA (enhancer repeats are indicated by the open rectangles), and the lower line represents the human repeat. Only regions upstream of the gene promoter are shown. Right-pointing arrows indicate transcription from the gene promoter, giving rise to pre-rRNA. The spacer promoter has been clearly identified for the mouse but its location has not yet been mapped accurately for the human RNA. The chromatin organization surrounding the CTCF binding site (indicated by a lollipop) that is most proximal to the gene promoter, is strikingly similar in both mouse and human. In both organisms, the CTCF binding sites are embedded within a CpG island (as predicted with EMBOSS-CpG Plot [57]; the length of the CpG domains is indicated below the respective rDNAs). Immediately upstream of the CTCF binding site, mouse rDNA chromatin is enriched in 'active' histone modifications. A surprisingly similar result was previ [file 1756-8935-3-19-S6.PDF]

Figure S6 - van de Nobelen et al

A

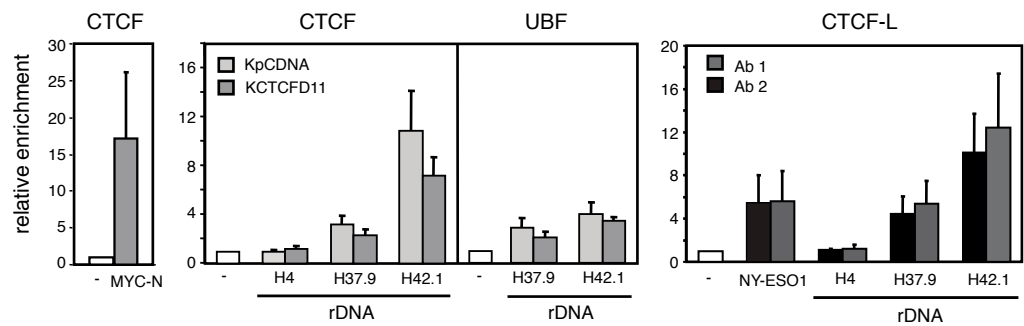

B

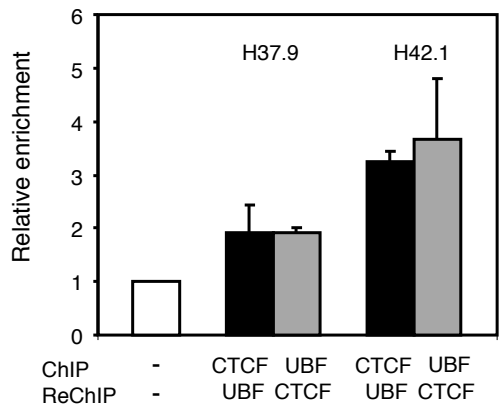

C

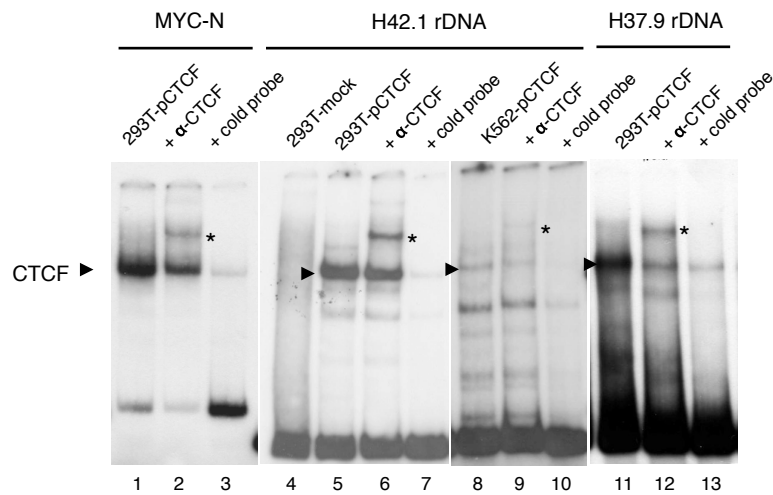

Supplement: Additional file 7 — Figure S6: CCCTC binding factor (CTCF) and CTCFL interact with human ribosomal (r)DNA in vivo. (A) Chromatin immunoprecipitation (ChIP) analysis on human rDNA. ChIP analysis with CTCF, CTCFL (two independent antibodies (Abs) were used) and UBF antisera, showing binding to the IGS of the rDNA repeat (sites H4, H37.9 and H42.1). Chromatin was prepared from non-transfected K562 cells or from cells stably transfected with CTCF (KCTCFD11) or the empty vector (KpCDNA). Relative enrichment was quantified by real-time PCR with the indicated primer sets. Known CTCF (MYC-N) and CTCFL (NY-ESO1) target sites were used as positive control for ChIP. Data were normalized against the enrichment for the negative control MYC-H.1. The value for the amount of PCR product present from the ChIP assay without antibody was set as 1 (white bars). Error bars represent the SEM of five to seven independent experiments for CTCF, eight to 10 for upstream binding factor (UBF), and four for CTCFL. (B) Sequential ChIP (ChIP-reChiP) analysis on human rDNA. Primary ChIP was performed as above, and CTCF or UBF ChIP products were subjected to a second immunoprecipitation (reChIP) with anti-UBF or anti-CTCF antisera, respectively. Relative enrichment was quantified by real-time PCR with primers for H37.9 or H42.1 rDNA, and data were normalized as in part (A). Error bars represent SEM of three independent experiments. Results show in vivo binding of CTCF and UBF simultaneously at rDNA sites. (C) CTCF interacts with human rDNA in vitro. Electrophoretic mobility shift assay (EMSA) analysis with nuclear extracts from 293T cells or K562 cells transfected with CTCF or mock transfected. 32P-labeled PCR fragments of MYC-N (positive control), H42.1 rDNA and H37.9 rDNA were used as probes. Unlabeled (cold) probes were used as competitors (Myc-N = 90% competition (compare lanes 1 and 3); H42.1 = 95% competition (compare lanes 5 and 7); H37.9 = 85% competition (compare lanes 11 and 13). Arrowheads indicate binding [file 1756-8935-3-19-S7.PDF]

Figure S7 - van de Nobelen et al

A

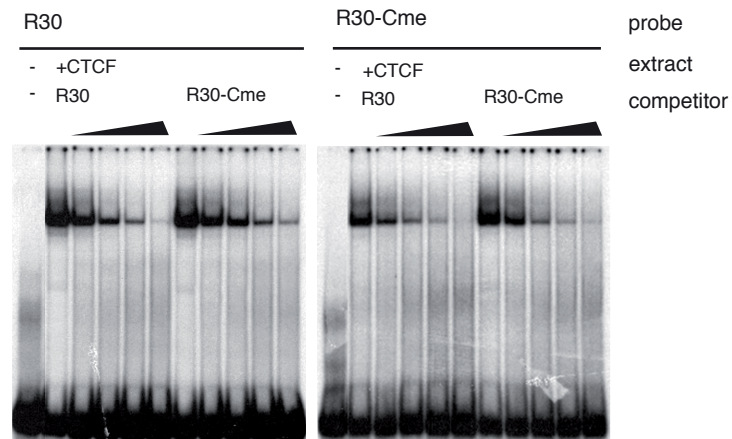

B

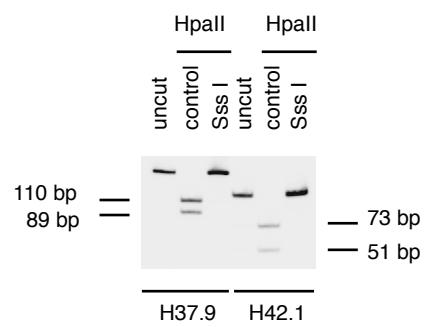

Supplement: Additional file 8 — Figure S7: CCCTC binding factor (CTCF) binds human ribosomal (r)DNA in a methylation-sensitive manner. (A) Influence of methylation on the binding of CTCF to mouse rDNA. Band-shifts were performed using the mouse rDNA probe (R30) and the same probe methylated on two cytosine residues (R30-CmE). Competition was assessed by adding increasing amounts of unlabeled probe. (B) Control and SssI methyltransferase-treated H37.9 and H42.1 rDNA probes were digested with the methylation-sensitive enzyme HpaII to assess the level of in vitro methylation. Fragments were separated in 8% polyacrylamide gels. [file 1756-8935-3-19-S8.PDF]

Figure S8 - van de Nobelen et al

A

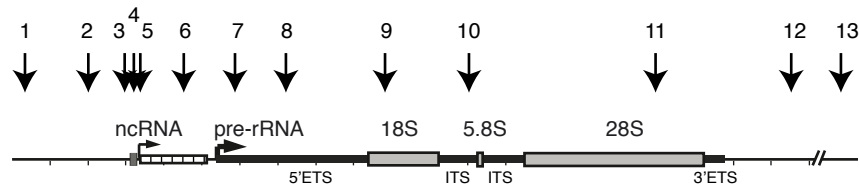

B

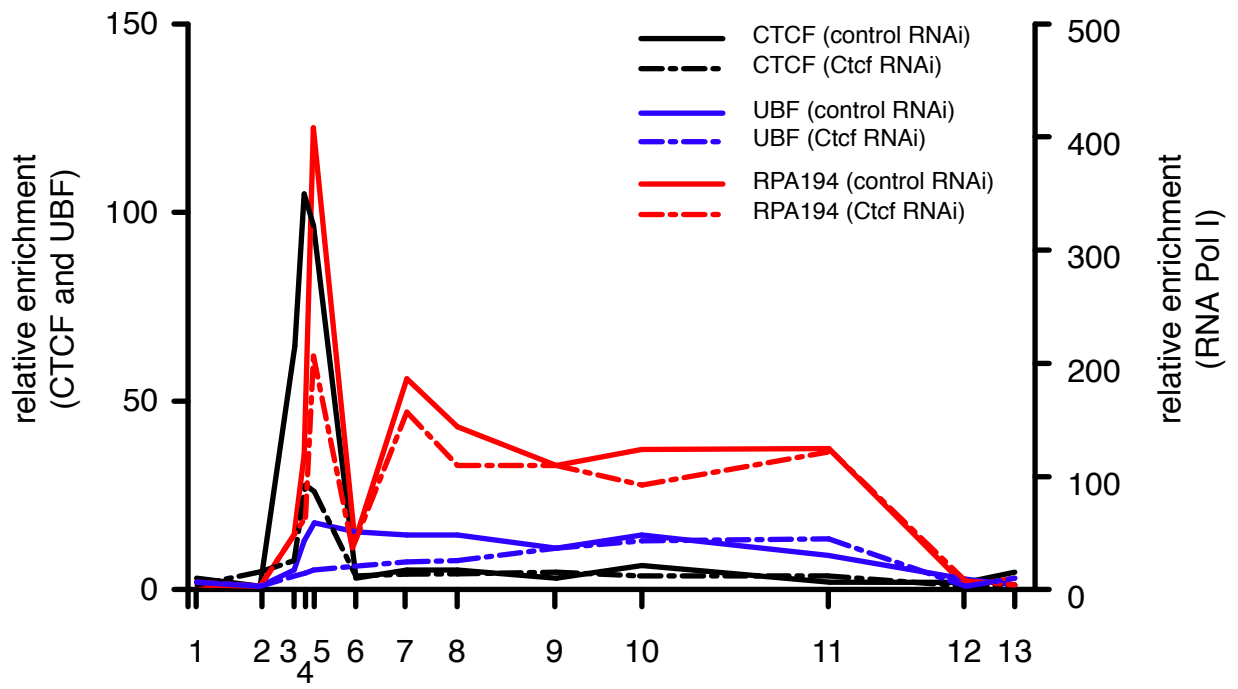

Supplement: Additional file 9 — Figure S8: Chromatin immunoprecipitation (ChIP) analysis in embryonic stem (ES) cells. (A) Outline of mouse rDNA repeat. The position of the primer pairs used in the ChIP in panel B is indicated by downward-pointing arrows. Transcription initiation from the spacer promoter (yielding ncRNA) and the gene promoter (yielding pre-rRNA) is indicated by right-pointing arrows. The 47S pre-rRNA is divided into 5' and 3' external transcribed spacer (ETS), internal transcribed spacers (ITS), and 18S, 5.8S and 28S rRNA genes. The approximate positions of the CCCTC binding factor (CTCF) consensus site (gray box) and enhancer repeats (white boxes) are indicated. (B) ChIP assay on mouse rDNA. Binding of CTCF (black), upstream binding factor (UBF) (purple/blue) and RNA polymerase I (red) to mouse rDNA was analyzed using the primer pairs indicated in part (A). Embryonic stem (ES) cells were treated with control (straight lines) or Ctcf (stippled lines) RNAi constructs. ES cell nuclei were fixed with 1% formaldehyde, and protein-DNA complexes were immunoprecipitated with antibodies against the indicated proteins. Upon depletion of CTCF, binding of both RNA Pol I and UBF was diminished. Strikingly, for both proteins, loss in binding was greatest near the CTCF binding site, strongly suggesting an important role for CTCF in the binding of these proteins at or near the spacer promoter (RNA Pol I, UBF) and on the enhancer repeat (UBF). The fact that RNA Pol I binding was not affected at or downstream of the gene promoter is consistent with previous data. [file 1756-8935-3-19-S9.PDF]

Figure S9 - van de Nobelen et al

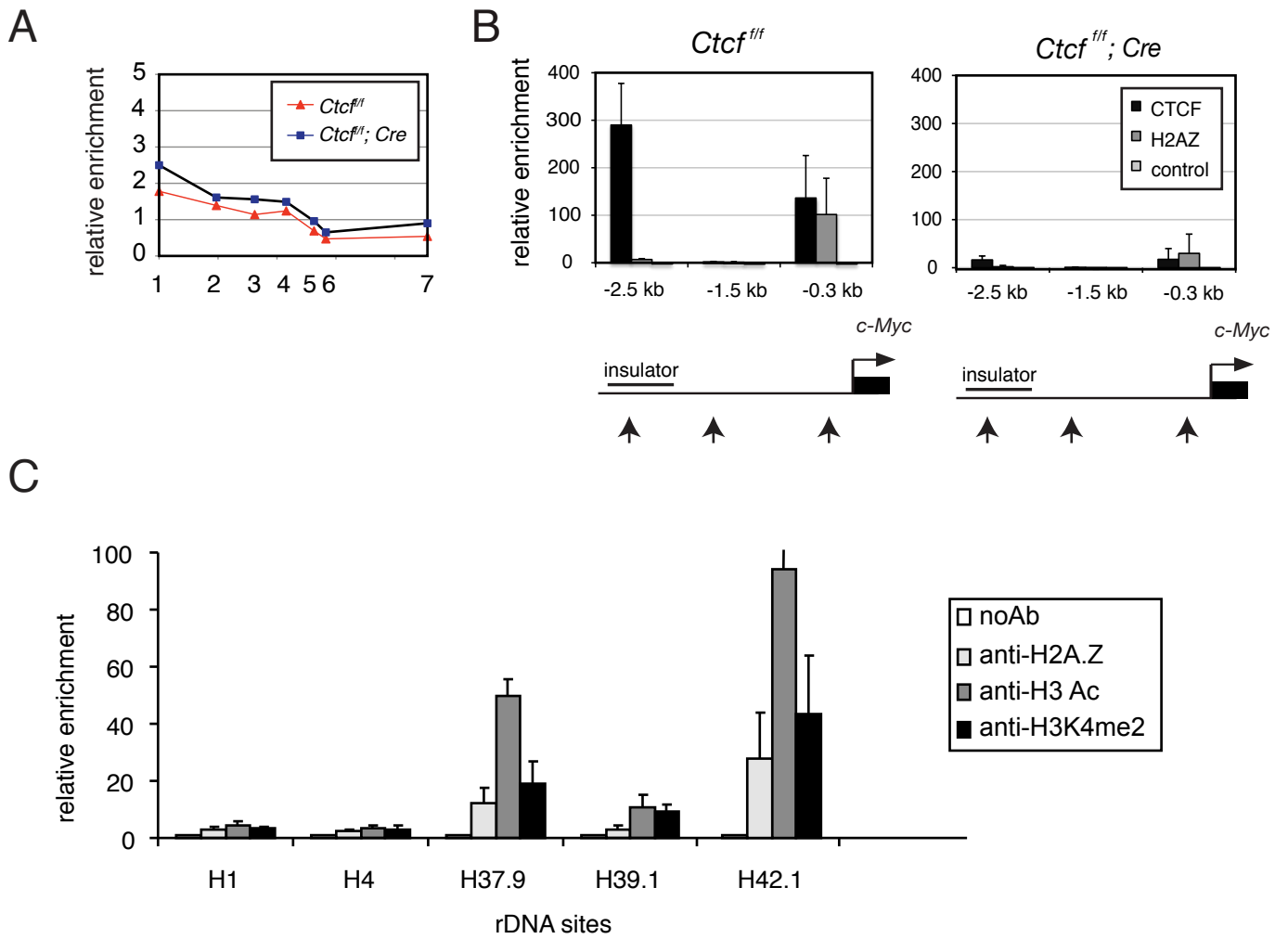

Supplement: Additional file 10 — Figure S9: CCCTC binding factor (CTCF) regulates histone deposition. (A) Binding of histone H3 to mouse ribosomal (r)DNA. Chromatin immunoprecipitation (ChIP) analysis is the same as shown in Figure 6, but a lower y-axis scale is used to demonstrate the histone H3 binding pattern. Enrichment was normalized to input and is shown relative to the Amylase gene (note that in this case the Amylase gene is not a negative control, because histone H3 will also bind this gene, hence the 'low' relative enrichment). Histone H3 was distributed in a similar manner in Cre-treated Ctcff/f mouse embryonic fibroblasts (MEFs) compared with non-treated cells. Interestingly, binding appeared to diminish as the ribosomal gene promoter area is approached. This might be due to the fact that active ribosomal genes contain fewer nucleosomes [12]. (B) Binding of CTCF and H2A.Z to the c-Myc gene. ChIP analysis was performed as in Figure 6C, using the regulatory region upstream of the c-Myc transcriptional start site (SD of three independent experiments indicated). The position of the primer sets is indicated with arrows. (C) Binding of modified and variant histones to human rDNA. ChIP analysis was performed as in Figure S6A (see Additional file 7). Chromatin was prepared from K562 cells. Protein-DNA complexes were immunoprecipitated with antibodies against the indicated proteins. ChIP analysis showed specific binding of H2A.Z, H3ac and H3K4me2 to sites H37.9 and H42.1 of the rDNA. [file 1756-8935-3-19-S10.PDF]

Figure S10- van de Nobelen et al

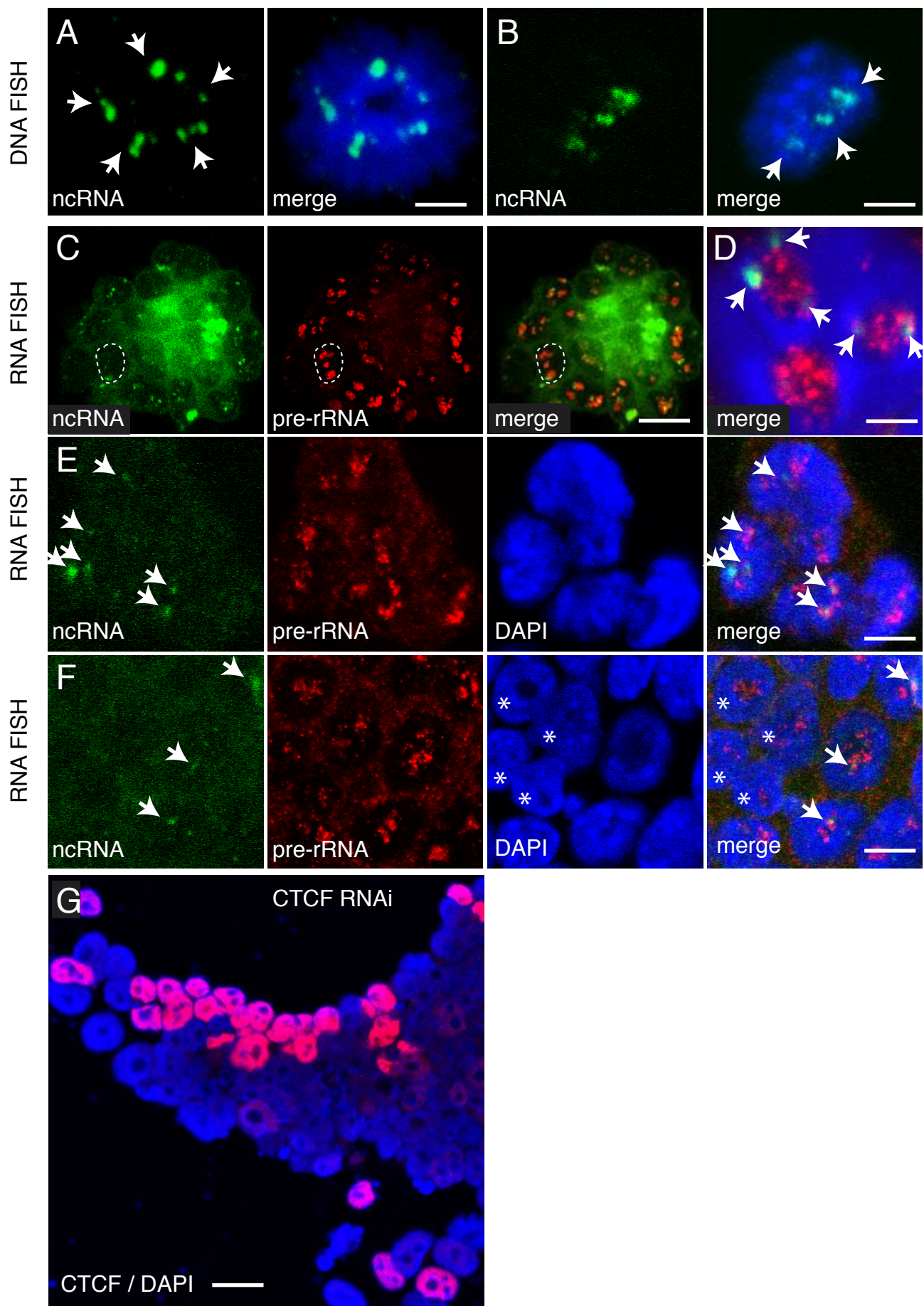

Supplement: Additional file 11 — Figure S10: Spatial segregation of non-coding (nc)RNA and pre-rRNA transcription. A, (B) DNA fluorescent in situ hybridization (FISH) analysis. The ncRNA probe (biotin-labeled, green) was hybridized to fixed and denatured ES cells. (A) Cell in prometaphase, with chromosomes condensed but not yet aligned. The ncRNA probe localized in distinct spots (arrows) adjacent to the strongly 4',6-diamidino-2-phenylindole (DAPI)-stained centromeric DNA. (B) An interphase cell, with the ncRNA probe localized to the nucleolus (visualized as weakly staining DAPI regions). Scale bars = (A) 2 μm, (B) 3 μm. (C-F) RNA FISH analysis. The ncRNA probe (biotin-labelled, green) and pre-rRNA probe (digoxygenin-labeled, red) were hybridized to fixed non-denatured ES cells. (C-E) Embryonic stem (ES) cells contain normal levels of CCCTC binding factor (CTCF), whereas (F) ES cells transfected with a pSUPER plasmid have CTCF knockdown. (C) Low resolution image of a small ES cell colony (cells in the middle are less well visualized because these cells grow in clumps). Multiple nuclei (one is outlined), particularly on the edge of the colony, had readily detectable ncRNA and pre-rRNA signals. Scale bar = 10 μm. (D) High resolution confocal image of a single DAPI-stained ES cell nucleus. Both ncRNA and pre-rRNA signals were localized exclusively to the three nucleoli present within this cell. Five ncRNA spots are visible (arrows), localized at the periphery of the nucleoli. Scale bar = 1 μm. (E, F) Confocal images taken with similar settings. (E) non-treated ES cells; (F) CTCF RNAi-treated ES cells. The ncRNA signal is indicated by arrows. Depletion of CTCF led to a reduction in ncRNA. ncRNA was lacking in many cells throughout a 3D confocal stack. In cells lacking ncRNA, pre-rRNA levels also seemed to be affected (see asterisks). Scale bars (E, F) = 8 μm. (G) Knock-down of CTCF in ES cells. ES cells were transfected with a pSUPER plasmid to knock down CTCF. After 4 days, < 50% of the cells express [file 1756-8935-3-19-S11.PDF]
