# Supplementary material for: CTCF regulates the local epigenetic state of ribosomal DNA repeats
Source: Epigenetics Chromatin. 2010 Nov 8;3:19. doi: 10.1186/1756-8935-3-19 (PMC2993708; doi:10.1186/1756-8935-3-19)
Supplement: Additional file 2 — Table S1: Mass spectrometry results for biotin tagged CTCF (CTCF-bio). [file 1756-8935-3-19-S2.DOC]

Additional File 2.

*Table S1.Mass spectrometry results for CTCF-bio.*

| Identified Protein | Molecular weight (kDa) | Acc. Number | Mascot Score* | Identified Peptides* |
| --- | --- | --- | --- | --- |
| UBF | 97 | gi 136653 | 110, 85 | 2, 2 |
| RNA pol I (RPA116) | 116 | gi 38614338 | 38 | 1 |
| RNA pol I (RPA194) | 194 | gi 2330007 | 210, 294 | 6, 8 |
| RNA pol I (RPA40) | 40 | gi 120538451 | 124 | 1 |
| PAF49 | 49 | gi 38602694 | 165, 213 | 4, 6 |
| PAF53 | 53 | gi 12328816 | 115, 108 | 2, 2 |
| WDR5 | 36 | gi 14250247 | 47 | 1 |
| CTCF | 80 | gi 6681073 | 69, 102 | 2, 2 |

* When two numbers are listed the data are from two independent mass spectrometry experiments. Proteins listed above were not detected in control samples (cells expressing BirA only).
