# Supplementary material for: CTCF regulates the local epigenetic state of ribosomal DNA repeats
Source: Epigenetics Chromatin. 2010 Nov 8;3:19. doi: 10.1186/1756-8935-3-19 (PMC2993708; doi:10.1186/1756-8935-3-19)

Figure S2 - van de Nobelen et al

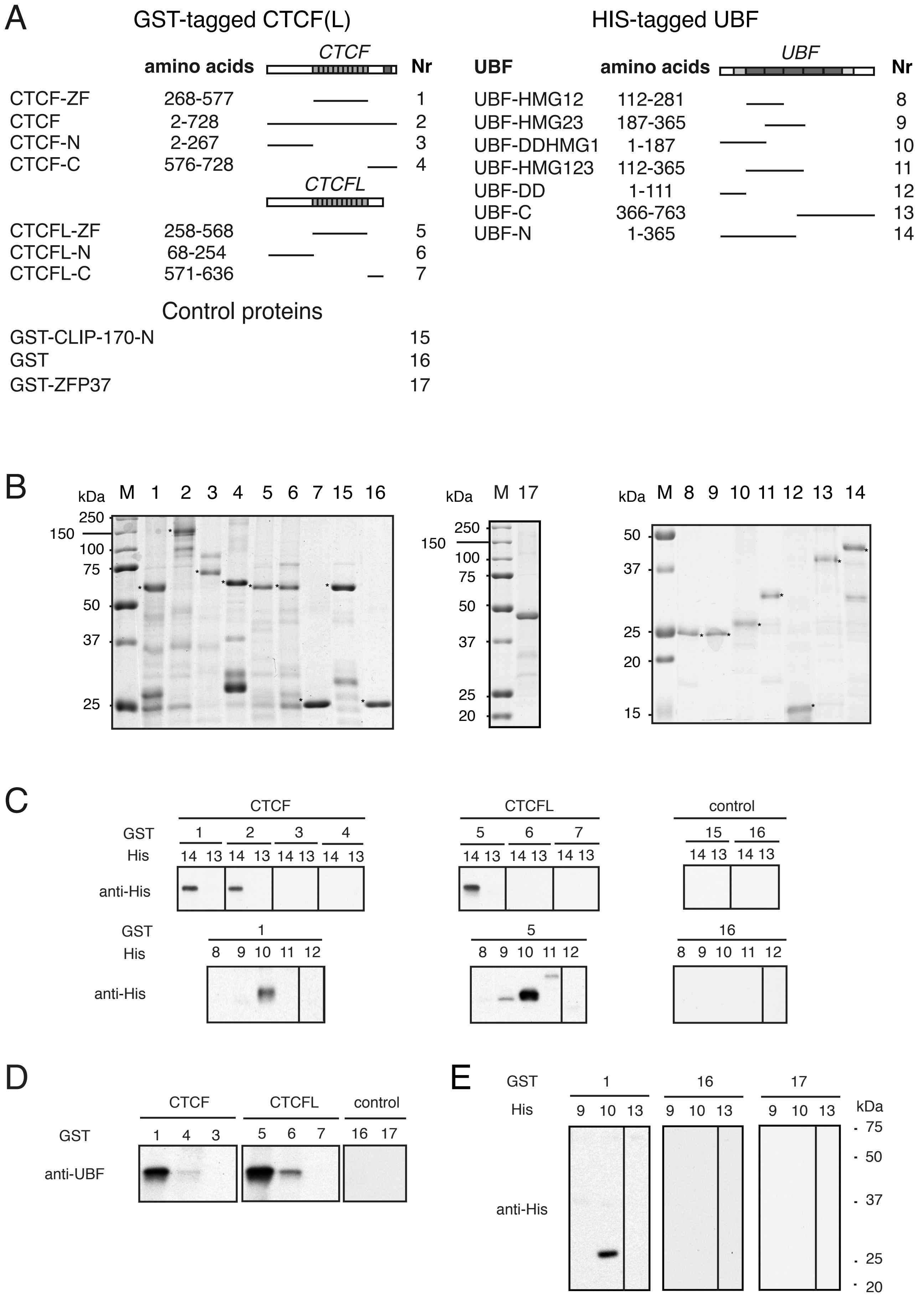

Supplement: Additional file 3 — Figure S2: Direct interaction of CCCTC binding factor (CTCF) and CTCFL with UBF. (A) Schematic representation of the glutathione-S-transferase (GST)- and histidin (His)-tagged fusion proteins used. (B) Expression of GST- and His-tagged fusion proteins. Proteins were expressed in bacteria and affinity purified. Fusion proteins are indicated by asterisks. (C) Interaction between bacterially produced proteins. Purified GST- and His-tagged fusion proteins were incubated together, followed by GST pull-down. Western blots were incubated with an anti-His antibody. The experiments revealed a direct interaction between the CTCF and CTCFL zinc finger (ZF) domains and the upstream binding factor (UBF) dimerization domain plus high mobility group (HMG)-box 1. His-tagged proteins containing either the dimerization domain of UBF or HMG-box 1 only weakly bound CTCF and CTCFL, indicating that both regions are necessary for efficient interaction. (D) Bacterially produced CTCF and CTCFL interacted with UBF derived from embryonic stem (ES). GST pull-down assays of bacterially produced CTCF and CTCFL mutants were performed with nuclear protein extracts from ES cells. Equal amounts of GST fusion proteins were incubated with nuclear extracts from ES cells. Binding was performed under low-salt conditions, and washing was performed under more stringent conditions. Western blots were incubated with an antibody against UBF to detect ES cell-derived UBF. GST-tagged CTCF and CTCFL were both able to pull down specifically UBF. The ZF domains of CTCF (1) and CTCFL (5) displayed prominent interaction with ES cell-derived UBF. (E) Bacterially produced ZFP37 did not interact with histidine (His)-tagged UBF. The ZF domain of murine ZFP37 a protein that is enriched in the nucleolus [55] was tagged with GST. To provide further evidence for the specificity of the CTCF-UBF interaction, we examined whether this ZF domain interacts with UBF. Purified GST-tagged ZFP37 was incubated with His-tagged UBF (con [file 1756-8935-3-19-S3.PDF]
