# Supplementary material for: CTCF regulates the local epigenetic state of ribosomal DNA repeats
Source: Epigenetics Chromatin. 2010 Nov 8;3:19. doi: 10.1186/1756-8935-3-19 (PMC2993708; doi:10.1186/1756-8935-3-19)
Supplement: Additional file 12 — Table S2: Primers used for band-shift assays [58]. [file 1756-8935-3-19-S12.DOC]

Additional File 12.

*Table S2.* Primers used for bandshifts.

| **name** | **sequence (5’ to 3’)** |
| --- | --- |
| R30s | TGTATGGTTGATCGAGACCATTGTCGGGCGACACCTAGTGGTGACAAGTTTCGGGAACGCTCCAGGCCTCT |
| R30as | AGAGGCCTGGAGCGTTCCCGAAACTTGTCACCACTAGGTGTCGCCCGACAATGGTCTCGATCAACCATACA |
| R30mut-s | TGTATGGTTGATCGAGACCATTGTCGGGCAATACCTAGTAGTGACAAGTTTCGGGAACGCTCCAGGCCTCT-3’ |
| R30metss^1)^ | TGTATGGTTGATCGAGACCATTGT[5Me-dC]GGG[5Me-dC]GACACCTAGTGGTGACAAGTTTCGGGAACGCTCCAGGCCTCT |
| R30 metas^1)^ | AGAGGCCTGGAGCGTTCCCGAAACTTGTCACCACTAGGTGT[5Me-dC]GCC[5Me-dC]GACAATGGTCTCGATCAACCATACA |
| F1 _F^2)^ | CTAGATGAAGAAATTGAGACCTCTACTGGATAGCTATGGTATTTACGTGTCTA |
| F1_B^2)^ | AGCTTAGACACGTAAATACCATAGCTATCCAGTAGAGGTCTCAATTTCTTCAT |

1) Ordered at OPERON Biotechnologies, Germany.

2) From the chicken lysozyme gene [58].
