# Supplementary material for: CTCF regulates the local epigenetic state of ribosomal DNA repeats
Source: Epigenetics Chromatin. 2010 Nov 8;3:19. doi: 10.1186/1756-8935-3-19 (PMC2993708; doi:10.1186/1756-8935-3-19)
Supplement: Additional file 13 — Table S3: Primers used for genotyping. [file 1756-8935-3-19-S13.DOC]

Additional File 13.

Table S3. Primers used for genotyping.

| **name** | **sequence (5’ to 3’)** |
| --- | --- |
| Probe 1F | TCCTGCCTCTGTCCAGTCAGAGA |
| Probe 1B | GCAGATCACTGTGTGTTCAAGGC |
| Probe 2F | CGAATGCCACCTTTGACTCTACC |
| Probe 2B | AAGCCTCGTCCTTCCGAGCCT |
| Rosa26 F (265) | GTGTAACTGTGGACAGAGGAG |
| Rosa26 F (266) | GAACTTGATGTGTAGACCAGG |
| BirA_F (91) | TTCAGACACTGCGTGACT |
| BirA_B (92) | GGCTCCAATGACTATTTGC |
| CTCFGB1 | AGCAAAAGCAAAACCAGGTTA |
| CTCFGF14 | AGGAGCCAGATGCCGAGCCTG |

Genotyping yields fragments of 350 bp (Rosa26), 514 bp (BirA), 599 bp (*Ctcf^bio^*), and 549 bp (wild type allele).
