# Supplementary material for: CTCF regulates the local epigenetic state of ribosomal DNA repeats
Source: Epigenetics Chromatin. 2010 Nov 8;3:19. doi: 10.1186/1756-8935-3-19 (PMC2993708; doi:10.1186/1756-8935-3-19)
Supplement: Additional file 14 — Table S4: Primers used for mouse chromatin immunoprecipitation (ChIP). [file 1756-8935-3-19-S14.DOC]

Additional File 14.

*Table S4. Primers used for mouse* ChIP.

| **name** | **sequence (5’ to 3’)** |
| --- | --- |
| Enh4_F (APS1) -4736 | GTCACCATTCTGCACTTGCAA |
| Enh4_B (APS1) -4584 | ACATGTGCATGGCAGCCATCTTG |
| Enh5_F -3736 | GTGTGTTTGTGCTCTATCTGCTG |
| Enh5_B -3641 | CACTTATTCTCAGGAGCTGCATG |
| Enh6_F -3088 | GTGAGTTCCAGGACTTACCAGAG |
| Enh6_B -2988 | CTGTGTAGCCCTATCGGACTTG |
| Enh3_F -2561 | CACTGCTTAGATGCTCCCTTCC |
| Enh3_B -2446 | ATCGTTCTTGAAGTCAAAGTACGTC |
| Enh2_F (spacer prom.) -2087 | AGGAGGCCGGGCAAGCA |
| Enh2_B (spacer prom.) -1975 | CGTACAGCAACTCGGTCTGCT |
| Enh_F (enhancer repeat) -1882 | CCTCCAGAAGCCCTCTCTTGTCCC |
| Enh_B (enhancer repeat) – 1779 | CAGCTGGCCGAGCCACACCGG |
| Prom_UCE_F -162 | AGTTGTTCCTTTGAGGTCCGGT |
| Prom_UCE_B -52 | GAGACAGGGAGGAAAGTGACAG |
| ChIP1s | ACCTCACTATGACCGGCTGAGATTC |
| ChIP1a | CCACCCGTAATGAGATCTGATGTCC |
| ChIP2s | ATGTGCCACCACTGCCCGGACTGA |
| ChIP2a | CACCTTTAACCTTAGGCAAATTTTA |
| ChIP3s | CACTGCTTAGATGCTCCCTTCC |
| ChIP3a | CACTGCTTAGATGCTCCCTTCC |
| ChIP4s | TGTTCGGGCGGGACGATG |
| ChIP4a | AGGTGTCGCCCGACAATG |
| ChIP5s | TGACAGGAGGCCGGGCAAGCA |
| ChIP5a | GCGTACAGCAACTCGGTCTGCT |
| ChIP7s | GTCACCCGGGGCGCTTGTACTTCTGAT |
| ChIP7a | TCGTGTCCTCTAGGCCTCAGATGTAA |
| ChIP9s | CCAAGTGTTCATGCCACGTG |
| ChIP10s | GCGCAGCGTTTGCTCTCT |
| ChIP10a | CACACAAGCCGAGCCACAT |
| ChIP11s | GCTTGTCTCAAAGATTAAGCCATGC |
| ChIP11a | TATTAGCTCTAGAATTACCACAGTT |
| ChIP12s | CCGGCTTGCCCGATTTCCGCGGGT |
| ChIP12a | GCCAGCAGGAACGAAACG |
| ChIP13s | GTAACTATGACTCTCTTAAGGTAGCCA |
| ChIP13a | CTTCACCGTGCCAGACTAGAG |
| ChIP14s | TGGTTGCTGGGATTTGAACTC |
| ChIP14a | CAGAGAAATACTGTCTCAGAAG |
| ChIP15s | ACTTGCAAACCGGGCCACTAAA |
| ChIP15a | TTCCTTGTTCTGTCACTCGGTTGC |
| amylase 99 | CTCCTTGTACGGGTTGGT |
| amylase 100 | AATGATGTGCACAGCTGAA |
| Myc_ChIP_1F (cMyc 1,5kb downstr prom.) | GCTCCTAAACCAGAGTCTGCTG |
| Myc_ChIP_1B (cMyc 1,5kb downstr prom.) | CATACACCTCCACACAGTTCCAG |
| Myc_ChIP_2F (cMyc promoter) | TGACTCGCTGTAGTAATTCCAGC |
| Myc_ChIP_2B (cMyc promoter) | TCTCACTCCAGAGCTGCCTTC |
| Myc_5’INS_F (cMyc insulator) | CAGAACCTGGAAACCCTGCAG |
| Myc_5’INS_B (cMyc insulator) | GTTGTGGCTCTCGGATTTGTG |
| 3’HS1_529_F (3’HS1) | AATCAGTGGAACACTTCTGC |
| 3’HS1_530_B (3’HS1) | GTCTCAGGTTGTCAACTAAAGC |
