# Supplementary material for: CTCF regulates the local epigenetic state of ribosomal DNA repeats
Source: Epigenetics Chromatin. 2010 Nov 8;3:19. doi: 10.1186/1756-8935-3-19 (PMC2993708; doi:10.1186/1756-8935-3-19)
Supplement: Additional file 15 — Table S5: Primers used for northern blotting and nuclear run-on assays. [file 1756-8935-3-19-S15.DOC]

Additional File 15.

*Table S5. Primers used for northern blot and* nuclear run-on.

| **name** | **sequence (5’ to 3’)** |
| --- | --- |
| **Northern blot** | |
| 5’ETS_F | GTTCCTATTGGACCTGGAGA |
| 5’ETS_B | CGGTTGGAATGGTGGAGCCA |
| GAPDH_F | TGAACGGGAAGCTCACTGG |
| GAPDH_B | TCCACCACCCTGTTGCTGTA |
| **Run-on : promoter probe (290 bp; 129bp overlap transcript)** | |
| rDNAprom_F - 161 | GTTGTCAGGGTCGACCAGTTGT |
| IGS_F_B +129 | GACAGCTTCAGGCACCGC |
| **Run-on: spacer promoter probe (361bp; 219bp overlap transcript)** | |
| IGSB_F -2140 | CAGGTTGGTGACACAGGAGAG |
| Enh_B - 1779 | CAGCTGGCCGAGCCACACCGG |
| **Run-on: actin and histone H3 probes** | |
| Actin-exon5-F | ATCATGTTTGACACCTTCAACACC |
| Actin-exon5-B | GAACCGCTCGTTGCCAATAGT |
| Histone H3i-F | AAGCAGCTGGCCACCAAG |
| Histone H3i-B | CTCCTGCAGAGCCATGACG |
