# Supplementary material for: CTCF regulates the local epigenetic state of ribosomal DNA repeats
Source: Epigenetics Chromatin. 2010 Nov 8;3:19. doi: 10.1186/1756-8935-3-19 (PMC2993708; doi:10.1186/1756-8935-3-19)
Supplement: Additional file 16 — Table S6: Primers used for real-time PCR on embryonic stem (ES) cell ribosomal (r)RNA. [file 1756-8935-3-19-S16.DOC]

Additional File 16.

*Table S6. Primers used for real-time PCR on ES cell rRNA*.

| **name** | **sequence (5’ to 3’)** |
| --- | --- |
| ncrRNA2s (ChIP7s) | GTCACCCGGGGCGCTTGTACTTCTGAT |
| ncrRNA2a (ChIP7a) | TCGTGTCCTCTAGGCCTCAGATGTAA |
| ncrRNA1s  (IGSB_F -2140) | CAGGTTGGTGACACAGGAGAG |
| ncrRNA1a  (Enh_B - 1779) | CAGCTGGCCGAGCCACACCGG |
| 45S forward | GACACGCTGTCCTTTCCCTA |
| 45S reverse | AGGCTGGACAAGCAAAACAG |
