# Supplementary material for: CTCF regulates the local epigenetic state of ribosomal DNA repeats
Source: Epigenetics Chromatin. 2010 Nov 8;3:19. doi: 10.1186/1756-8935-3-19 (PMC2993708; doi:10.1186/1756-8935-3-19)
Supplement: Additional file 17 — Table S7: Primers used for human chromatin immunoprecipitation (ChIP) and band-shift assays [59-61]. [file 1756-8935-3-19-S17.DOC]

Additional File 17.

*Table S7. Primers used for human ChIP and band-shifts.*

| name | sequence (5’-3’) | Human rDNA coordinate site |
| --- | --- | --- |
| MYC-N_F^1)^  MYC-N_B | ACAAGGAGGTGGCTGGAAAC  TTCCCCTCCTGGCTTTTAGT |  |
| MYC-H.1_F^2)^  MYC-H.1_B | CAACGCAACACAGGATATGG  TTCCCCTCCTGGCTTTTAGT |  |
| NY-ESO1_F^3)^  NY-ESO1_B | ACCCGCAACCCACCCCACAC  GGGGCAGGCCTCTAACTGGG |  |
| H42.1 rDNA_F^4)^  H42.1 rDNA_B | GCTTCTCGACTCACGGTTTC  CCGAGAGCACGATCTCAAA | 42012-42031  42117-42135 |
| H37.9 rDNA_F^4)^  H37.9 rDNA_B | CCCTGGTCGATTAGTTGTGG  GTGCTCCCTTCCTCTGTGAG | 37818-37837  37997-38016 |
| H4 rDNA_F^5)^  H4 rDNA_B | CGACGACCCATTCGAACGTCT  CTCTCCGGAATCGAACCCTGA | 3990-4010  4072-4092 |

1) See ref [59].

2) See ref [60].

3) See ref [61].

4) for the position of the CTCF binding sites H37.9 and H42.1 in the IGS of the human rDNA repeat, see Figure S5 (additional File 6).

5) See ref [15].
